# Navitoclax enhances the effectiveness of EGFR-targeted antibody-drug conjugates in PDX models of EGFR-expressing triple-negative breast cancer

**DOI:** 10.1186/s13058-020-01374-8

**Published:** 2020-11-30

**Authors:** Jason J. Zoeller, Aleksandr Vagodny, Veerle W. Daniels, Krishan Taneja, Benjamin Y. Tan, Yoko S. DeRose, Maihi Fujita, Alana L. Welm, Anthony Letai, Joel D. Leverson, Vincent Blot, Roderick T. Bronson, Deborah A. Dillon, Joan S. Brugge

**Affiliations:** 1 Department of Cell Biology and Ludwig Center at Harvard, Harvard Medical School, 240 Longwood Avenue, Boston, MA 02115 USA; 2 Department of Medical Oncology, Dana-Farber Cancer Institute, Boston, MA USA; 3 Department of Pathology, Brigham & Women’s Hospital, Boston, MA USA; 4 Department of Oncological Sciences, Huntsman Cancer Institute, University of Utah, Salt Lake City, UT USA; 5 Oncology Development, AbbVie, North Chicago, IL USA; 6 Department of Pathology, Harvard Medical School, Boston, MA USA

**Keywords:** BCL-2, BCL-X_L_, ABT-263, Navitoclax, Apoptosis, TNBC, ADC, EGFR, Cytotoxic, PDX

## Abstract

**Background:**

Targeted therapies for triple-negative breast cancer (TNBC) are limited; however, the epidermal growth factor receptor (EGFR) represents a potential target, as the majority of TNBC express EGFR. The purpose of these studies was to evaluate the effectiveness of two EGFR-targeted antibody-drug conjugates (ADC: ABT-414; ABBV-321) in combination with navitoclax, an antagonist of the anti-apoptotic BCL-2 and BCL-X_L_ proteins, in order to assess the translational relevance of these combinations for TNBC.

**Methods:**

The pre-clinical efficacy of combined treatments was evaluated in multiple patient-derived xenograft (PDX) models of TNBC. Microscopy-based dynamic BH3 profiling (DBP) was used to assess mitochondrial apoptotic signaling induced by navitoclax and/or ADC treatments, and the expression of EGFR and BCL-2/X_L_ was analyzed in 46 triple-negative patient tumors.

**Results:**

Treatment with navitoclax plus ABT-414 caused a significant reduction in tumor growth in five of seven PDXs and significant tumor regression in the highest EGFR-expressing PDX. Navitoclax plus ABBV-321, an EGFR-targeted ADC that displays more effective wild-type EGFR-targeting, elicited more significant tumor growth inhibition and regressions in the two highest EGFR-expressing models evaluated. The level of mitochondrial apoptotic signaling induced by single or combined drug treatments, as measured by DBP, correlated with the treatment responses observed in vivo. Lastly, the majority of triple-negative patient tumors were found to express EGFR and co-express BCL-X_L_ and/or BCL-2.

**Conclusions:**

The dramatic tumor regressions achieved using combined agents in pre-clinical TNBC models underscore the abilities of BCL-2/X_L_ antagonists to enhance the effectiveness of EGFR-targeted ADCs and highlight the clinical potential for usage of such targeted ADCs to alleviate toxicities associated with combinations of BCL-2/X_L_ inhibitors and systemic chemotherapies.

## Background

The epidermal growth factor receptor (EGFR) [1] is a potential target for therapeutic intervention, as it is highly expressed in the majority of TNBC [2–9]. To date, clinical responses to EGFR-targeted receptor tyrosine kinase inhibitors and function-blocking antibodies have been disappointing [1, 10]; however, these trials were designed without selection for EGFR-expressing triple-negative patient tumors and limited by dose-limiting EGFR-associated toxicities. Thus, it remains unclear whether EGFR is an actionable and relevant target and whether it can be exploited safely. Next-generation tumor-specific EGFR-targeted antibodies (ABT-806) [11] and their antibody-drug conjugates (Table 1) [12–14] represent promising alternative approaches because these agents enable tumor-selective EGFR-targeting as well as subsequent delivery of the cytotoxic payload independent of EGFR inhibition, and eliminate the systemic side effects associated with exposures to conventional anti-EGFR agents and cytotoxic chemotherapy. ABT-414 (depatux-m), comprising ABT-806 conjugated to the cytotoxic monomethyl auristatin F (MMAF), has demonstrated single-agent activities across several tumor types in which *EGFR* is amplified, mutated (e.g., EGFRvIII mutations) or over-expressed [12]. Next-generation antibody-drug conjugates (ADCs) comprising an affinity-matured version of ABT-806 conjugated to either monomethyl auristatin E (ABBV-221) [13] or the ultra-potent pyrrolobenzodiazepine (PBD) dimer (ABBV-321) [14] exhibit enhanced EGFR affinities and killing potencies and have demonstrated effectiveness within EGFR-expressing tumors or tumor cell lines. Combined, the higher affinity of ABBV-321 for EGFR and potency of the PBD payload is predicted to permit targeting and killing of cells with lower receptor expression. Given the elevated expression of EGFR in TNBC, we were interested in investigating the efficacy of the EGFR-targeted ADCs in TNBCs.

**Table 1:** EGFR-targeted and non-targeted antibody-drug conjugates

ADC	Antibody	Linker	Payload
ABT-414	ABT-806	mc (non-cleavable)	MMAF
AB095-MMAF	AB095	mc (non-cleavable)	MMAF
ABBV-321	ABT-806 AM1	mc-VC (cleavable)	PBD
AB095-PBD	AB095	mc-VC (cleavable)	PBD

We previously demonstrated that inhibition of the pro-survival proteins BCL-2 and BCL-X_L_ via ABT-263/navitoclax dramatically enhanced the effectiveness of a HER2-targeted ADC (T-DM1) [15]. The success of navitoclax+T-DM1 prompted us to explore whether navitoclax can enhance the effectiveness of either ABT-414 or ABBV-321 in a series of EGFR-expressing patient-derived xenografts (PDX) of TNBC [16].

## Methods

### Patient-derived xenografts

Patient-derived xenograft models of TNBC were developed and provided by Y.D., M.F., and A.W. (Huntsman Cancer Institute). As previously described [17], tumor fragments were orthotopically transplanted into female NOD.scid mice (Charles River Labs; RRID:IMSR_ARC:NODSCID). For the PDX studies presented within Figs. 2, 3, and 4 and Supplemental Figure 7, each mouse was transplanted with one tumor fragment in order to generate one tumor per mouse. Procedures were completed in accordance with IACUC#0990 and Harvard University ARCM policies.

### Drug treatments

100 mpk ABT-263/navitoclax (AbbVie) was administered p.o. once per day. ABT-263 was formulated according to AbbVie recommendations in 60% phosal 50 PG (Lipoid), 30% polyethylene glycol 400 (DOW Chemical), and 10% ethanol. 10 mpk ABT-414 (AbbVie) or the non-tumor targeted ADC AB095-MMAF (AbbVie) was administered i.p. once per week; 0.5 mpk ABBV-321 (AbbVie) or the non-tumor targeted ADC AB095-PBD (AbbVie) was also administered i.p. once per week. ADCs were prepared in 0.9% sterile saline for injection (Hospira). The vehicle-treated mice received the vehicles corresponding to drug-treated mice. Tumor-bearing mice were randomized according to pre-treatment tumor volumes. For the studies presented within Figs. 2, 3, and 4 and Supplemental Figure 7, tumor volumes prior to treatment initiation are presented in Supplemental Figure 2, Supplemental Figure 3, Supplemental Figure 4, Supplemental Figure 6, and Supplemental Figure 7. For the studies presented within Figs. 3 and 4 and Supplemental Figure 7, the number (*n*) of tumor-bearing animals randomized per group were in agreement with the results from our initial in vivo studies (Fig. 2), which determined statistical significance between vehicle- and combo-treated PDX using *n* = 3–6 tumor-bearing animals per group. For the initial studies (Fig. 2), the 14-day treatment plan was based upon our previous work which demonstrated the efficacy of navitoclax and a HER2-targeted ADC (T-DM1) within the same time frame [15]. Pre-treatment body weights were used for individualized mpk calculations. Body weights measured at the experimental endpoints were used to calculate percent reductions in body weight.

### Specimen collection

For tumor-bearing animals, tumors were collected at the experimental endpoints, bisected along the longitudinal axis and fixed in 10% neutral buffered formalin (Sigma). For nontumor-bearing animals, blood was collected from the retro-orbital sinus < 4 h post-treatment on day 17 (experimental endpoint). The serum was isolated (BD Microtainer SST) and stored at − 20 **°**C. Carcasses were collected at the experimental endpoint, dissected along the midline and fixed in Bouin’s solution (Sigma).

### H&E

Formalin-fixed paraffin-embedded (FFPE) tumors and Bouin’s-fixed paraffin-embedded livers were prepared, processed, sectioned, and H&E-stained by the Harvard Rodent Histopathology Core.

### Clinical chemistry

Serum alanine aminotransferase (ALT), aspartate aminotransferase (AST), alkaline phosphatase (ALP), and albumin (ALB) levels were measured by Charles River Laboratories, Clinical Pathology Services (Shrewsbury, MA).

### FISH

FISH assays were performed using bacterial artificial chromosome clones RP11-148P17 and RP11-81B20 (Oakland Children’s Hospital) to construct probes for a 333-kb region including *EGFR*. Probes were biotin-labeled using the Random Prime DNA Labeling System (Invitrogen) according to the manufacturer’s protocol and detected with rhodamine-streptavidin. A FITC-labeled probe to the chromosome 7 centromeric region (CEP7) was purchased from Abbott/Vysis. The specificity of probe binding was verified using normal lymphocyte metaphase spreads. Dual-color FISH was performed on whole tissue sections. Slides were counterstained with DAPI/Antifade (Vector Labs) and evaluated using an Olympus BX51 fluorescence microscope. Hybridization signals were scored in ≥ 20 tumor cells for each case. The average *EGFR* signals per cell, average CEP7 signals per cell and, *EGFR*:CEP7 ratio were calculated. Previously described *EGFR* copy number classifications were applied [9, 18].

### IHC

EGFR (Cell Signaling Technologies 4267), BCL-2 (Dako M0887), and BCL-X_L_ (Cell Signaling Technologies 2764) IHC assays and *H*-scores [19] were performed as previously described [15, 20].

### Tumor volumes

For in vivo tumor measurements in 2-dimensions, caliper-based measurements approximated length (*l*) and width (*w*). These values were used to calculate volume via ½(*l* × *w*^2^). For ex vivo tumor measurements in 3-dimensions, caliper-based measurements approximated length, width, and height (*h*). These values were used to calculate volume via $$ \pi \times \raisebox{1ex}{$1$}\!\left/ \!\raisebox{-1ex}{$6$}\right.\left(l\times w\times h\right) $$. For each tumor, tumor growth was calculated as a percentage of pre-treatment tumor volume and based upon pre- and post-treatment in vivo tumor volumes. Tumor-bearing mice that died before the experimental endpoints were excluded from volume comparisons; however, tumor data for these mice is presented in the Additional file 1.

### Microscopy

Histology slides were visualized and images were acquired via a Nikon Eclipse E200 microscope, Idea color camera, and SPOT software for MAC.

### Statistics

GraphPad Prism for MAC was utilized for statistical tests (Welch’s *t* test) and correlation analyses (Pearson’s *r*).

### Microscopy-based dynamic BH3 profiling

HCI-010 or HCI-025 patient-derived xenografts were dissociated using a combination of mechanical and enzymatic disruption methods. PDX were cut into small pieces using a scalpel and added to digestion medium composed of DMEM/F12 (Gibco) with DNase I recombinant grade I (130 U/ml, Roche), hyaluronidase from bovine testes (0.1 KU/ml, Sigma), and collagenase type I from rat tail (2 mg/ml, Gibco). To obtain a single-cell suspension, tumor samples were incubated in a dissociation medium at 37 °C with continuous shaking for 45–90 min. Freshly isolated PDX-derived tumor cells were cultured in advanced DMEM/F12 (Gibco) with N2 supplement (Gibco), B27 supplement (Gibco), 1% l-glutamine (Gibco), and 10% FBS (Gibco) at 37 °C under 5% CO_2_. For DBP, PDX-derived tumor cells were seeded in 384 well plates (Corning 3764BC) and treated for 24 h with ABT-263 (AbbVie), ABBV-321 (AbbVie), ABT-263+ABBV-321, AB095-PBD (AbbVie), or ABT-263+AB095-PBD. DBP was performed as previously described [21, 22]. Briefly, BIM peptide dilution curves were generated using untreated tumor cells. A concentration of BIM peptide that corresponded to 10% of cytochrome c-negative cells was selected based upon the dilution curves and was used to perform BH3 profiling on the drug-treated tumor cells. Twenty-four hours post-treatment, the tumor cells were washed three times with PBS using the BioTek 406EL plate washer. Consequently, the BIM peptide was added using the HP D300e Digital Dispenser (Hewlett-Packard Development Company) at the designated concentration. Tumor cells were incubated in a BH3 profiling buffer containing 0.002% digitonin for 1 h. Tumor cells were fixed in paraformaldehyde for 15 min. The fixative was subsequently neutralized using a Tris/Glycine buffer. Tumor cells were stained overnight with Hoechst 33342 (tumor nuclei, Invitrogen), anti-cytochrome c Alexa Fluor 647-conjugated antibody (BioLegend), anti-cytokeratin antibody (tumor cells, BioLegend), and anti-vimentin antibody (tumor cells, BioLegend). Prior to imaging, the stain solution was removed via washes using the BioTek 406EL plate washer (BioTek). Fluorescence microscopy images from DBP plates were acquired using the IXM XLS high-content widefield microscope (ICCB Longwood Screening Facility, Harvard Medical School). Analysis of the images was performed using the MetaMorph Microscopy Automation and Image Analysis Software. The Multi Wavelength Cell Scoring Module was used to quantify the fraction of cytochrome c-positive tumor cells per well. All subsequent data analysis was performed in Microsoft Excel or GraphPad Prism.

### TMA

Tissue microarrays (TMA) corresponding to patient triple-negative breast cancers (HTMA-240) were obtained from the Breast Biorepository at the DFCI/HCC. HTMA-240 samples were previously collected via DFCI IRB#93085 and were de-identified prior to analysis. HTMA-240 was sectioned by the Brigham & Women’s Hospital Specialized Histopathology Core, and unstained FFPE were assayed for EGFR and co-expression of BCL-2/X_L_ according to the IHC methods described above. *H*-scores were determined by breast pathologist Dr. Deborah Dillon (BWH) according to standard procedures [19] and using reference controls [EGFR, MDA-MB-468 cell-derived xenografts; BCL-2, human tonsil tissue; BCL-X_L_, HCI-025 patient-derived xenografts].

## Results

### PDX models of TNBC express EGFR, BCL-2, and BCL-X_L_

Seven previously generated patient-derived xenograft models of HER2−/ER−/PR− breast cancer [16] were established and maintained via orthotopic transplantation in female NOD.scid mice [17]. Five models were derived from primary breast tumors that were either treatment naïve (HCI-002; HCI-004; HCI-016; HCI-019) or had undergone prior treatment (HCI-001), whereas two models (HCI-010; HCI-015) were derived from advanced metastatic breast cancers with multiple prior treatment exposures (Table 2).

**Table 2:** PDX models of triple-negative breast cancers

PDX	Patient source^a^	Patient treatment^b^	HER2 status^c^	ER status	PR status	EGFR *H*-score^d^	BCL-2 *H*-score^e^	BCL-X_L_ *H*-score
HCI-001	PT	+ Prior T×	**–**	**–**	**–**	188	10	100
HCI-002	PT	− Prior T×	**–**	**–**	**–**	121	230	90
HCI-004	PT	− Prior T×	**–**	**–**	**–**	76	10	220
HCI-010	PE	+ Prior T×	**–**	**–**	**–**	228	10	10
HCI-015	MT	+ Prior T×	**–**	**–**	**–**	193	20	60
HCI-016	PT	− Prior T×	**–**	**–**	**–**	90	280	90
HCI-019	PT	− Prior T×	**–**	**–**	**–**	208	40	155

^a^*PT* primary breast tumor, *PE* pleural effusion, *MT* metastatic breast tumor

^b^T×, treatment as previously described [16] and unpublished data kindly provided by A.W.

^c^HER2, ER, and PR as previously described [16] and unpublished data kindly provided by A.W.

^d^*H*-score represents the average of 4–6 tumors per PDX

^e^*H*-score represents one tumor surveyed per PDX kindly provided by A.W.

**Fig. 1:**
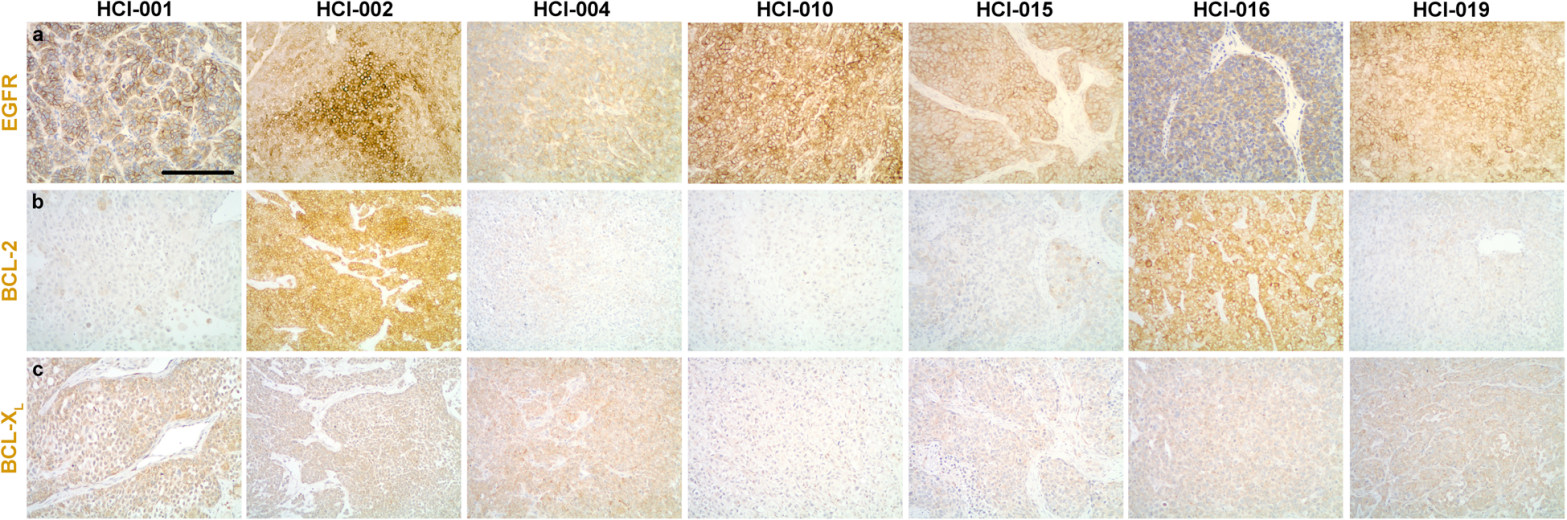
Comparison of EGFR, BCL-2, and BCL-X_L_ expression levels. Seven PDX models of TNBC were immuno-stained for EGFR (**a**), BCL-2 (**b**), and BCL-X_L_ (**c**). Representative immuno-stains are shown. The IHC results were semi-quantitated via *H*-score assessment and summarized in Table 2. Scale bar, ~ 200 μm

To compare EGFR, BCL-2, and BCL-X_L_ expression levels within these tumor models, we performed IHC assays and *H*-score (*H*) assessment for each protein (Fig. 1 and Table 2). These models displayed a range of EGFR expression levels (Fig. 1a), with HCI-004 (*H*_*x̅*_ = 76) as the lowest EGFR-expressing tumor model and HCI-010 (*H*_*x̅*_ = 228) as the highest EGFR-expressing tumor model. Two models (HCI-002 and HCI-016) were distinguished by high BCL-2 expression whereas the other five models expressed low levels of BCL-2 (Fig. 1b). There was also variation in the expression of BCL-X_L_, which ranged from *H*-scores of 10 to 220 (Fig. 1c).

To compare *EGFR* within these tumor models, we performed FISH assays and, as previously described [9], classified *EGFR* copy number alterations (Supplemental Table 1a). Based upon *EGFR*:CEP7 assessments (Supplemental Table 1b), none of the models examined was characterized by *EGFR*:CEP7 ≥ 2 and classified as *EGFR*-amplified; however, HCI-001, HCI-002, HCI-004, HCI-015, HCI-016, and HCI-019 were characterized by *EGFR* trisomy, and HCI-010 was characterized by *EGFR* polysomy (Supplemental Table 1b). Consistent with *EGFR* polysomy and EGFR expression (Fig. 1 and Table 2), the HCI-010 model was distinguished by the greatest proportion of tumor cells with ≥ 4 *EGFR* per cell (~ 26%). For the other PDX models evaluated, there was no obvious correlation between *EGFR* copy numbers and EGFR expression levels.

### Navitoclax enhances the in vivo efficacy of ABT-414 or ABBV-321

**Fig. 2:**
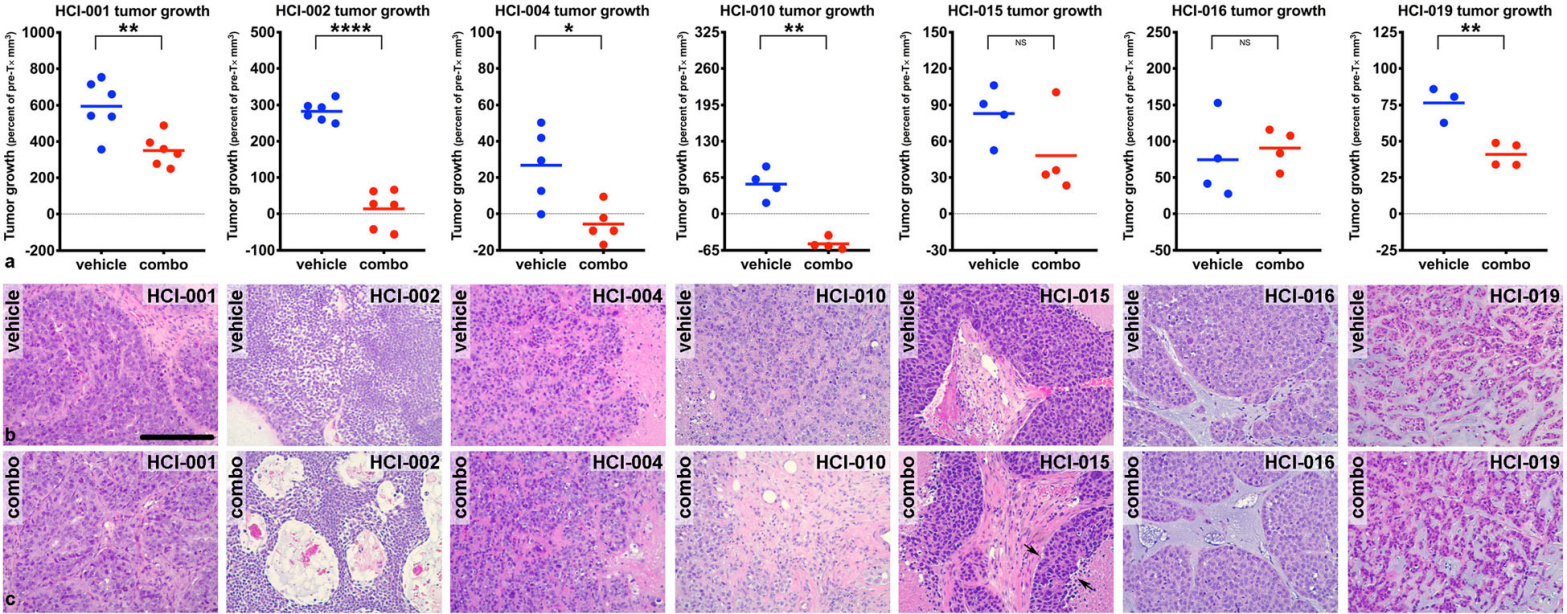
Evaluation of ABT-263/navitoclax+ABT-414 treatment responses. Seven EGFR-expressing PDX models were treated with ABT-263+ABT-414 [combo] or the vehicles for both agents [vehicle] according to the dose and schedule presented in Supplemental Figure 1a. Fourteen days post-treatment, significant tumor growth inhibition and regressions were observed in five of seven PDX models: HCI-001, HCI-002, HCI-004, HCI-010, and HCI-019 (**a**). The graphs present tumor growth as a percent of pre-treatment tumor volumes for each tumor (*n* = 3–6 per group). Each symbol represents a tumor. Each line represents the mean. Note that consistent and substantial regressions are restricted to HCI-010 tumors. *p* values < 0.05 (*), < 0.01 (**), < 0.001 (***), and < 0.0001 (****) are indicated. *p* values > 0.05 = NS. *p* values, determined by Welch’s one-tailed test, are presented for vehicle versus combo comparisons in Supplemental Table 2a. Representative H&E images for vehicle- (**b**) and combo-treated (**c**) tumors are shown to highlight treatment-associated pathological responses within HCI-002, HCI-010, and HCI-015 tumors. Note that HCI-002-treated tumors were characterized by regions of multi-focal cellular dropout; HCI-010-treated tumors were characterized by a desmoplastic stroma; HCI-015-treated tumors displayed reduced tumor cell content (→ ←) associated with expanded areas of necrosis. Scale bar, ~ 200 μm

To obtain an initial assessment of which PDX models were most sensitive to navitoclax+ABT-414 treatments, we evaluated the efficacy of this drug combination in all seven EGFR-expressing PDX models. For these studies, tumor-bearing mice (*n* = 4–6 per group) were randomized into one of two groups: one to be treated with navitoclax+ABT-414 and another with the vehicles for both agents (Supplemental Figure 1a). Navitoclax (100 mpk) was administered once per day and ABT-414 (10 mpk), once per week. Using caliper-based measurements, we calculated tumor volumes pre- (Supplemental Figure 2a) and post-treatment (Supplemental Figure 2b). Fourteen days post-treatment (Fig. 2), we observed significant reductions in tumor growth in five of seven combination-treated tumor models (HCI-001; HCI-002; HCI-004; HCI-010; HCI-019). Tumor regressions were observed in HCI-002, HCI-004, and HCI-010, with the most consistent and substantial tumor regressions being observed in HCI-010 tumors (Fig. 2a). Compared to the other PDX models, HCI-010 tumors were distinguished by the highest EGFR expression levels and the highest percentage of cells with ≥ 4 *EGFR* copies (Table 2 and Supplemental Table 1). Non-significant reductions in tumor growth were observed in the two additional combination-treated tumor models (HCI-015; HCI-016); however, these determinations could have been limited by sample size (Fig. 2a).

Macroscopic evaluation of H&E-stained tumor sections from the vehicle-treated and combo-treated tumors (Supplemental Figure 2c) supported the tumor growth inhibition observed within HCI-001, HCI-002, HCI-004, and HCI-010 tumors. Microscopic evaluation of the H&E sections corresponding to these tumors (Fig. 2b, c) also revealed pathological responses within HCI-002 and HCI-010 tumors as well as HCI-015 tumors. Although these responses were characterized by a reduction in viable invasive tumor cell content, each model exhibited unique treatment-associated histology. For example, HCI-002-treated tumors were characterized by regions of multi-focal cellular dropout whereas HCI-010-treated tumors were characterized by a desmoplastic stroma. Interestingly, HCI-015-treated tumors displayed reduced tumor cell content associated with expanded areas of necrosis.

**Fig. 3:**
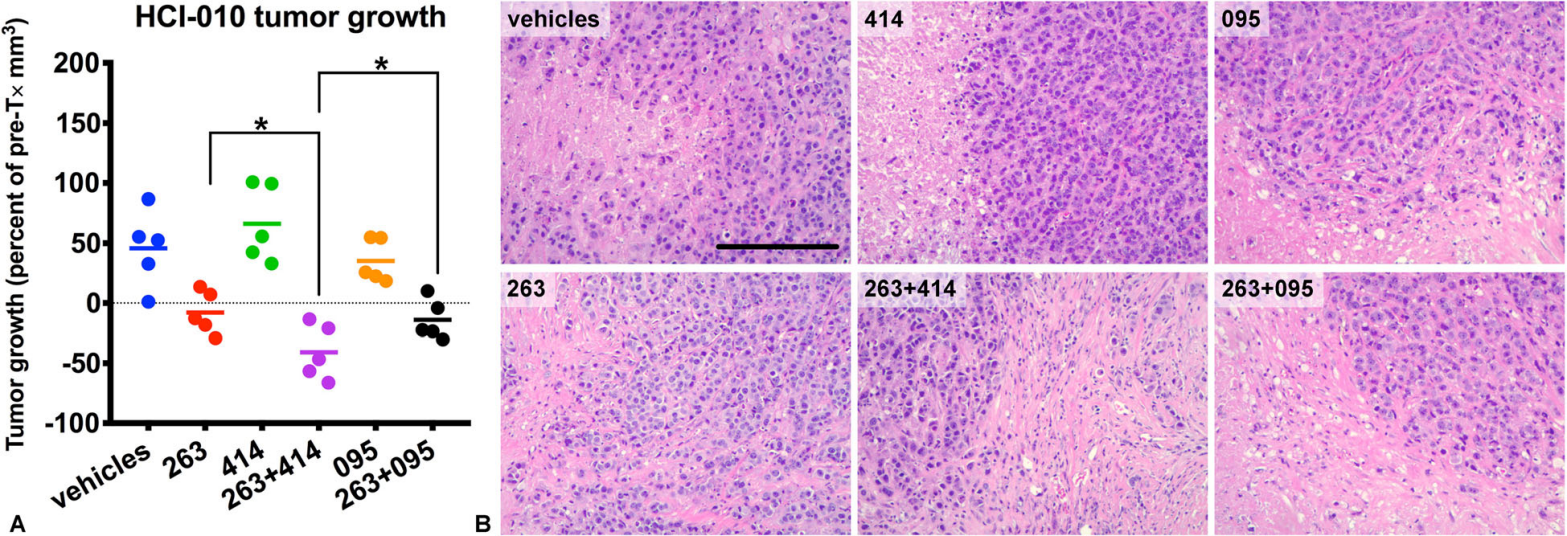
ABT-263/navitoclax enhances ABT-414 treatment responses within HCI-010 tumors. HCI-010 tumors were treated with vehicles, ABT-263, ABT-414, ABT-263+ABT-414, AB095-MMAF, or ABT-263+AB095-MMAF (Supplemental Figure 1b). Graphs present tumor growth as a percent of pre-treatment tumor volumes for each tumor (*n* = 5 per group). Each symbol represents a tumor. Each line represents the mean. Thirteen days post-treatment, significant tumor volume reductions and regressions were observed following either ABT-263 or ABT-263+ABT-414 treatments (**a**). Note greater regressions achieved after combined treatments. Note tumor growth was unaffected by ABT-414 or AB095-MMAF single agents. Note that ABT-263+AB095-MMAF was comparable to single-agent ABT-263. *p* values < 0.05 (*) are indicated for the most relevant comparisons (Welch’s one-tailed test: ABT-263 versus ABT-263+ABT-414 and ABT-263+ABT-414 versus ABT-263+AB095-MMAF). *p* values, determined by Welch’s one-tailed test, for vehicles versus ABT-263, ABT-414, ABT-263+ABT-414, AB095-MMAF, and ABT-263+AB095-MMAF, are presented in Supplemental Table 2b. Representative H&E images for tumors, corresponding to each treatment group, are shown (**b**) to highlight pathological responses associated with ABT-263 combined treatments. Scale bar, ~ 200 μm

Based upon our initial in vivo studies, which indicated that HCI-010 tumors were the most sensitive to combined navitoclax+ABT-414 treatments, we next compared single agents and combined treatments in the HCI-010 PDX model. As a control, we also included a non-tumor [tetanus toxoid] targeted ADC (AB095-MMAF) as a single agent or in combination with navitoclax. For these studies, tumor-bearing mice (*n* = 5 per group) were randomized into one of six groups: navitoclax, ABT-414, navitoclax+ABT-414, AB095-MMAF, navitoclax+AB095-MMAF, and vehicles (Supplemental Figure 1b). Navitoclax (100 mpk) was administered once per day, 5 days per week, and MMAF-loaded ADCs (10 mpk), once per week. Similar to the initial studies, we calculated tumor volumes pre- (Supplemental Figure 3a) and post-treatment (Supplemental Figure 3b). Single-agent ABT-414 treatment had no effect, whereas treatment with navitoclax reduced tumor volume and caused regressions of ~ 20% on average in a subset of treated mice (Fig. 3a and Supplemental Figure 3c). However, combined navitoclax+ABT-414 treatment induced significant tumor regressions of ~ 40% on average (Fig. 3a and Supplemental Figure 3c), similar to the findings for the combined treatment of HCI-010 presented in Fig. 2. Tumor size was unaffected by single-agent AB095-MMAF treatment, whereas tumors treated with navitoclax+AB095-MMAF were comparable in size to tumors treated with single-agent navitoclax. Microscopic evaluation of the H&E slides also revealed notable pathological responses associated with navitoclax treatments, characterized by a reduction in viable invasive tumor cell content (Fig. 3b). Together, these results provided evidence that navitoclax enhances the cytotoxicity of ABT-414.

**Fig. 4:**
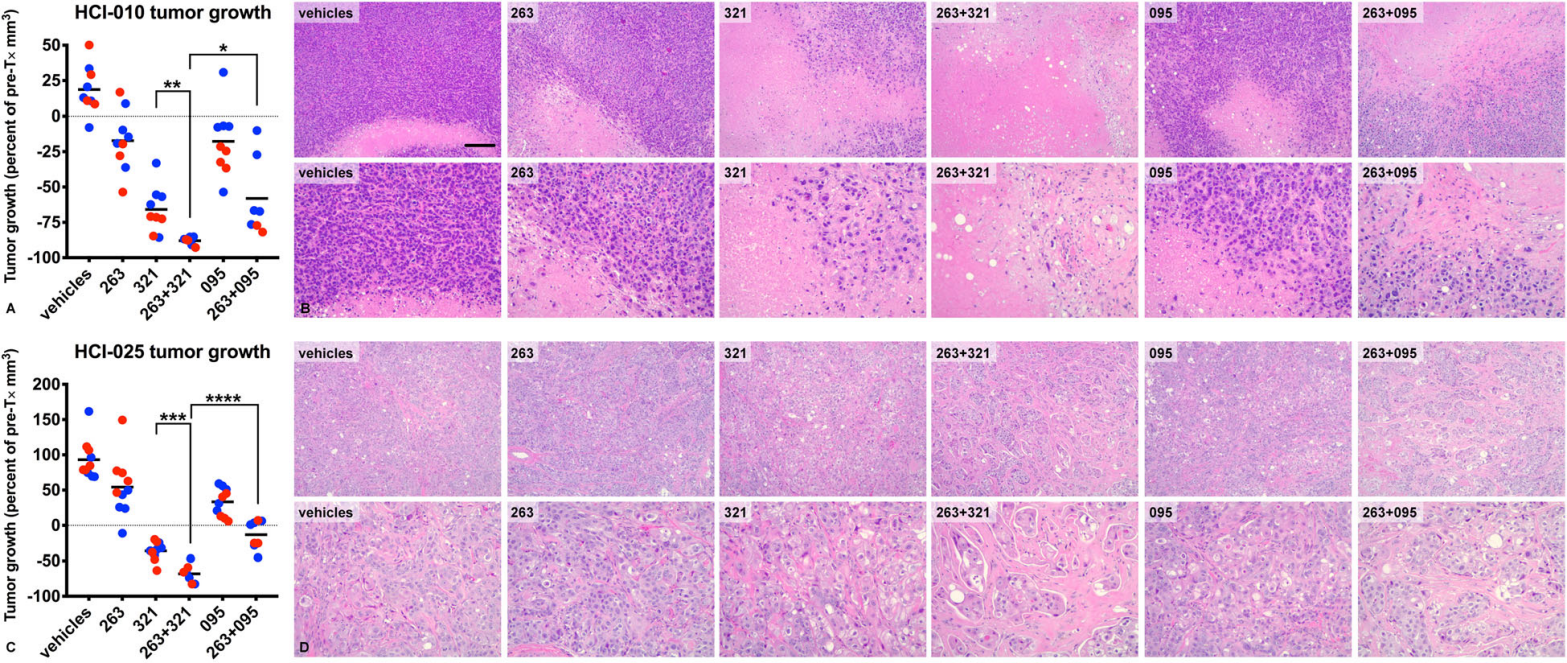
ABT-263/navitoclax enhances ABBV-321 treatment responses in vivo. HCI-010 (Supplemental Figure 1c) or HCI-025 (Supplemental Figure 1d) tumors were treated with vehicles, ABT-263, ABBV-321, ABT-263+ABBV-321, AB095-PBD, or ABT-263+AB095-PBD. The graphs present tumor growth as a percent of pre-treatment tumor volumes for each tumor (*n* = 2–5 per group) enrolled in one of two independent treatment studies (e.g., blue, study A; red, study B). Each symbol represents a tumor. Each line represents the grand mean. For HCI-010 (**a**), note the tumor volume reductions and regressions observed under ABT-263 treatment, and unlike ABT-414, under ABBV-321 treatment. Note the dramatic regressions observed following ABBV-321 are further enhanced by ABT-263 combined treatments. *p* values < 0.05 (*) and *p* values < 0.01 (**) are indicated for the most relevant comparisons (Welch’s one-tailed test: ABBV-321 versus ABT-263+ABBV-321 and ABT-263+ABBV-321 versus ABT-263+AB095-PBD). For HCI-025 (**c**) tumors, note the tumor volume reductions and regressions following ABBV-321 and enhanced responses following combined treatment with ABT-263. *p* values < 0.001 (***) and *p* values < 0.0001 (****) are indicated for the most relevant comparisons (Welch’s one-tailed test: ABBV-321 versus ABT-263+ABBV-321 and ABT-263+ABBV-321 versus ABT-263+AB095-PBD). *p* values, determined by Welch’s one-tailed test, for HCI-010 and HCI-025 vehicles versus ABT-263, ABBV-321, ABT-263+ABBV-321, AB095-PBD, and ABT-263+AB095-PBD are presented in Supplemental Table 2c. Representative H&E images for HCI-010 (**b**) or HCI-025 (**d**) tumors, corresponding to each treatment group, are shown to highlight pathological responses associated with ABBV-321 and ABT-263 treatments. Note that near-complete pathological responses were achieved following ABT-263+ABBV-321 combined treatments of HCI-010 tumors. The H&E are presented as matched low- and high-magnification images. Scale bar, ~ 200 μm

Since TNBCs are often enriched for EGFR expression in the absence of *EGFR* mutation or amplification, we also assessed an alternative EGFR-targeted ADC (ABBV-321) that displays an enhanced affinity for wild-type EGFR and carries the ultra-potent pyrrolobenzodiazepine (PBD) dimer [23] as an alternative cytotoxic payload. To evaluate the efficacy of ABBV-321 combined treatment in the HCI-010 model, tumor-bearing mice (*n* = 4–5 per group/2 studies) were randomized into six groups: navitoclax, ABBV-321, navitoclax+ABBV-321, AB095-PBD, navitoclax+AB095-PBD, and vehicles (Supplemental Figure 1c). 100 mpk of navitoclax was administered once per day, 5 days per week. Based upon ABBV-321’s enhanced EGFR affinity and potency, 0.5 mpk of PBD-loaded ADCs was administered once per week. We assessed tumor volumes pre- (Supplemental Figure 4a) and post-treatment (Supplemental Figure 4b). As observed in the previous experiments, navitoclax caused a reduction in HCI-010 tumor volume and induced partial tumor regression (Fig. 4a). However, unlike single-agent treatment with ABT-414, ABBV-321 single-agent treatment resulted in dramatic tumor regressions, on average ~ 66% (Fig. 4a). Notably, navitoclax enhanced the effectiveness of ABBV-321 as evidenced by dramatic and near-complete tumor regressions, on average ~ 88% (Fig. 4a). Macroscopic analysis of the H&E slides (Supplemental Figure 4d) indicated the tumor size reductions induced by ABBV-321 or navitoclax+ABBV-321 treatment. Consistent with these measurements, pathological responses characterized by the elimination of viable invasive carcinoma were associated with ABBV-321 and were dramatically enhanced by navitoclax (Fig. 4b).

We extended these studies to include an additional PDX (HCI-025). We selected HCI-025 after characterizing EGFR, BCL-2, and BCL-X_L_ expression levels in another seven PDX models of TNBC (Supplemental Table 3). HCI-025 was characterized by *EGFR* low polysomy (Supplemental Table 1) and comparable to HCI-010, high EGFR expression levels (*H* = 230) (Supplemental Figure 5). Compared to the HCI-010 model (Supplemental Figure 4), HCI-025 tumors expand at a faster rate in vivo (Supplemental Figure 6). To evaluate ABBV-321 combined treatment within the HCI-025 model, we performed a similar six-group treatment study (Supplemental Figure 1d) and calculated tumor volumes pre- (Supplemental Figure 6a) and post-treatment (Supplemental Figure 6b). HCI-025 tumors were sensitive to single-agent ABBV-321, displaying tumor regressions on average ~ 36% (Fig. 4c). As observed with HCI-010, navitoclax also enhanced the effectiveness of ABBV-321 in HCI-025 tumors, as evidenced by dramatic tumor regressions, on average ~ 68% (Fig. 4c). Consistent with these measurements, macroscopic analysis of the H&E slides highlighted ABBV-321-associated tumor size reductions (Supplemental Figure 6d). Moreover, pathological responses characterized by the elimination of viable invasive carcinoma were associated with combined navitoclax treatments (Fig. 4d).

AB095-PBD (non-tumor targeted ADC) and navitoclax+AB095-PBD also caused reductions in tumor volume and regressions in the HCI-010 and HCI-025 PDX models; however, responses to ABBV-321 alone were significantly greater than to AB095-PBD alone, and the responses to navitoclax+ABBV-321 were greater than navitoclax+AB095-PBD, supporting a contribution of EGFR-targeted effects to the efficacy of ABBV-321. These observations are consistent with some level of antigen-independent accumulation and uptake of AB095-PBD, which could be due to enhanced permeability and retention (EPR) of antibody-drug conjugates [24], tumor-associated macropinocytosis [25–28], or extracellular cleavage of the ADC linker [29]. These effects have been previously described for ADCs and other agents in vivo [25, 29–32].

To address combination effectiveness within the context of larger tumors, we evaluated treatment effects in the HCI-025 model after tumors had reached a volume of ~ 382.5 mm^3^ on average (Supplemental Figure 7). These pre-treatment tumor volumes were three times larger than the pre-treatment tumor volumes used in the experiments shown in Fig. 4 and Supplemental Figure 6a (~ 120 mm^3^ on average). Treatment groups and schedules were as described above (Supplemental Figure 1d). Significant ABBV-321 treatment effects, characterized by tumor growth arrest, were observed in mice carrying larger tumors (Supplemental Figure 7). Importantly, navitoclax once again enhanced the efficacy of ABBV-321 and resulted in moderate regressions characterized by ~ 21.5% on average (Supplemental Figure 7).

Adverse events were observed in HCI-010 and HCI-025 tumors treated with the PBD-loaded antibodies combined with navitoclax. Deaths were documented in two out of nine HCI-010 and three out of ten HCI-025 mice treated with navitoclax+ABBV-321 (Supplemental Figure 8a and e). Deaths were also documented in two out of nine HCI-010 and two out of ten HCI-025 mice treated with navitoclax+AB095-PBD (Supplemental Figure 8a and e). Interestingly, neither body weight reductions (Supplemental Figure 8b and f) nor tumor growth inhibition (Supplemental Figure 8c and d & g and h) distinguished HCI-010 or HCI-025 tumor-bearing mice that died. To provide additional insight into the nature of the toxicity, nontumor-bearing female NOD.scid mice (*n* = 5 per group) were treated with the PBD-loaded antibodies and or navitoclax according to the doses and schedules presented in Supplemental Figure 1d. Deaths were not observed in nontumor-bearing mice treated with PBD-loaded antibodies combined with navitoclax (Supplemental Figure 8i); however, body weight reductions (< 15%) were observed after day 12 (Supplemental Figure 8j-l). Seventeen days post-treatment, the serum was collected for clinical chemistry analyses, and carcasses were saved for necropsies. Microscopic evaluation of the livers revealed no obvious histopathology in any of the treatment groups except for those treated with PBD-loaded antibodies combined with navitoclax. Livers collected from animals treated with combined agents were distinguished by occasional apoptotic and or necrotic hepatocytes. To better assess liver function, we evaluated the serum samples for alanine aminotransferase (ALT; Supplemental Figure 8 m), aspartate aminotransferase (AST; Supplemental Figure 8n), alkaline phosphatase (ALP; Supplemental Figure 8o), and albumin (ALB; Supplemental Figure 8p). One of the five mice treated with either navitoclax+ABBV-321 (#16; Supplemental Figure 8 m and n) or navitoclax+AB095-PBD (#27; Supplemental Figure 8 m and n) displayed significantly elevated levels of ALT and AST (3.2-, 7.7-, 2.8-, and 4.2-fold higher than the mean of the vehicle-treated ALT and AST levels, respectively). None of the mice displayed significantly elevated levels of ALP (Supplemental Figure 8o), whereas there was a trend towards decreased levels of ALB in mice treated with combined agents (Supplemental Figure 8p). Together, these results suggest possible hepatotoxicity in some animals treated with combined agents.

### Early time point mitochondrial apoptotic signaling predicts treatment responses

**Fig. 5:**
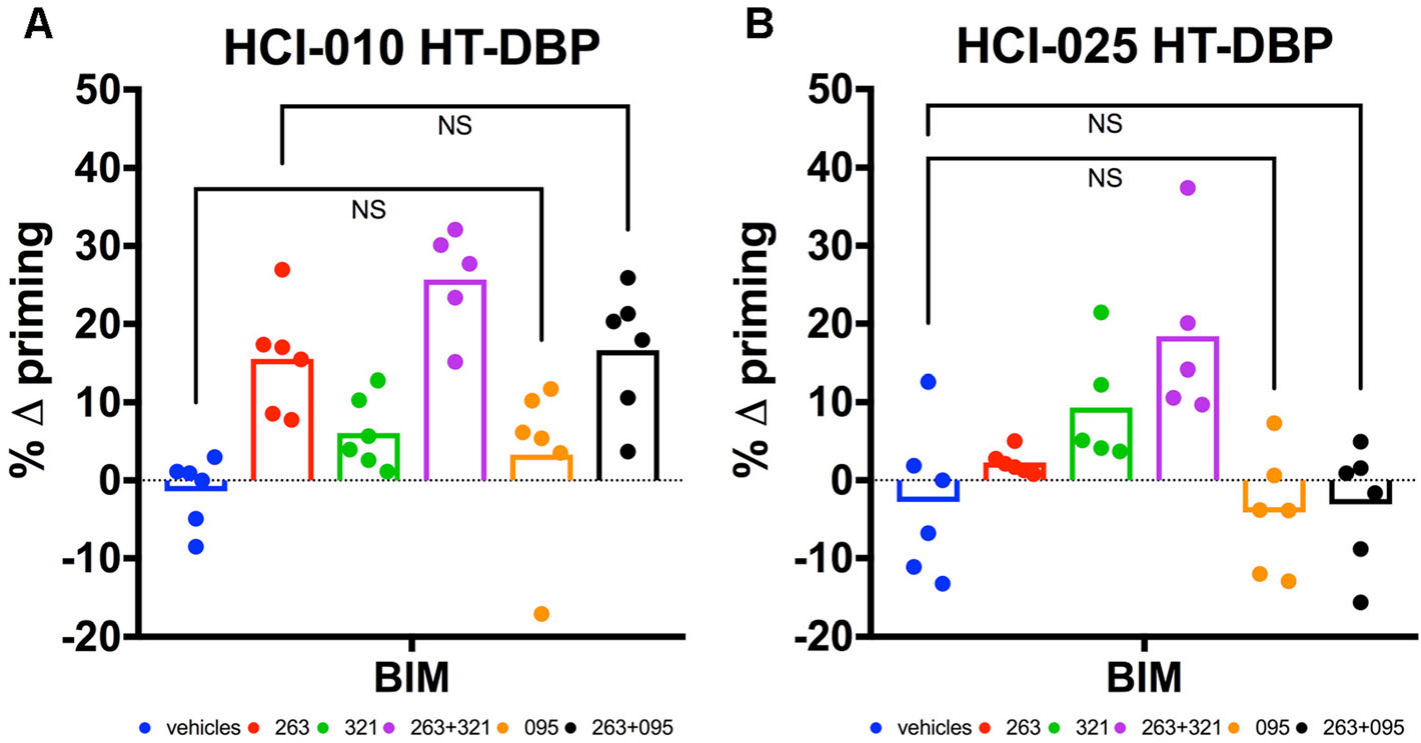
DBP predicts HCI-010 and HCI-025 treatment responses. HCI-010 (**a**) or HCI-025 (**b**) PDX-derived tumor cells were treated with vehicles, ABT-263, ABBV-321, ABT-263+ABBV-321, AB095-PBD, or ABT-263+AB095-PBD. The graphs present delta (Δ) priming as a normalized percentage (%) value for PDX-derived tumor cells (*n* = 3 tumors per PDX) evaluated in one of three independent DBP experiments. Each symbol represents PDX-derived cells evaluated once or twice per experiment. Each bar represents the mean. For HCI-010 (**a**), note that priming was observed under ABT-263 treatment and enhanced priming was observed under ABT-263+ABBV-321 combined treatments, consistent with the in vivo responses observed in Fig. 4. For HCI-010 (**a**), also note that insignificant priming was observed under either AB095-PBD or ABT-263+AB095-PBD treatment conditions. *p* values > 0.05 = NS are indicated for the most relevant comparisons (Welch’s one-tailed test: vehicles versus AB095-PBD and ABT-263 versus ABT-263+AB095-PBD). For HCI-025 (**b**), note that priming was observed under ABBV-321 treatment and enhanced priming following combined treatment with ABT-263, consistent with the in vivo responses observed in Fig. 4. For HCI-025 (**b**), also note that insignificant priming was observed under either AB095-PBD or ABT-263+AB095-PBD treatment conditions. *p* values > 0.05 = NS are indicated for the most relevant comparisons (Welch’s one-tailed test: vehicles versus AB095-PBD and vehicles versus ABT-263+AB095-PBD). *p* values, determined by Welch’s one-tailed test, for additional HCI-010 and HCI-025 comparisons are presented in Supplemental Table 2d

Dynamic BH3 profiling (DBP) [21, 22] was performed on PDX-derived tumor cells corresponding to HCI-010 (Fig. 5a) and HCI-025 (Fig. 5b) in order to assess the extent of mitochondrial apoptotic priming induced by ABBV-321 and/or ABT-263/navitoclax treatments. It was also of interest to assess whether AB095-PBD displayed apoptotic priming in vitro as this compound caused reductions in tumor size and regressions in vivo (Fig. 4). DBP revealed that both navitoclax and ABBV-321 single-agent treatments induced mitochondrial-associated apoptotic signaling within HCI-010 tumor cells, whereas only ABBV-321 single-agent treatments induced mitochondrial-associated apoptotic signaling within HCI-025 tumor cells. Notably, tumor cells derived from either HCI-010 or HCI-025 exhibited enhanced priming under combined navitoclax+ABBV-321 treatments in vitro. These responses were remarkably consistent with the treatment responses observed in vivo (Fig. 4). Surprisingly, treatments in vitro with AB095-PBD as a single agent failed to induce mitochondrial apoptotic signaling within either HCI-010 (Fig. 5a) or HCI-025 (Fig. 5b), and treatment with navitoclax+AB095-PBD in HCI-010 did not induce additional apoptotic priming relative to navitoclax alone, suggesting that the tumor reductions associated with this non-targeting ADC are dependent on factors within the tumor microenvironment in vivo.

### Triple-negative patient tumors co-express EGFR and BCL-2/X_L_

**Fig. 6:**
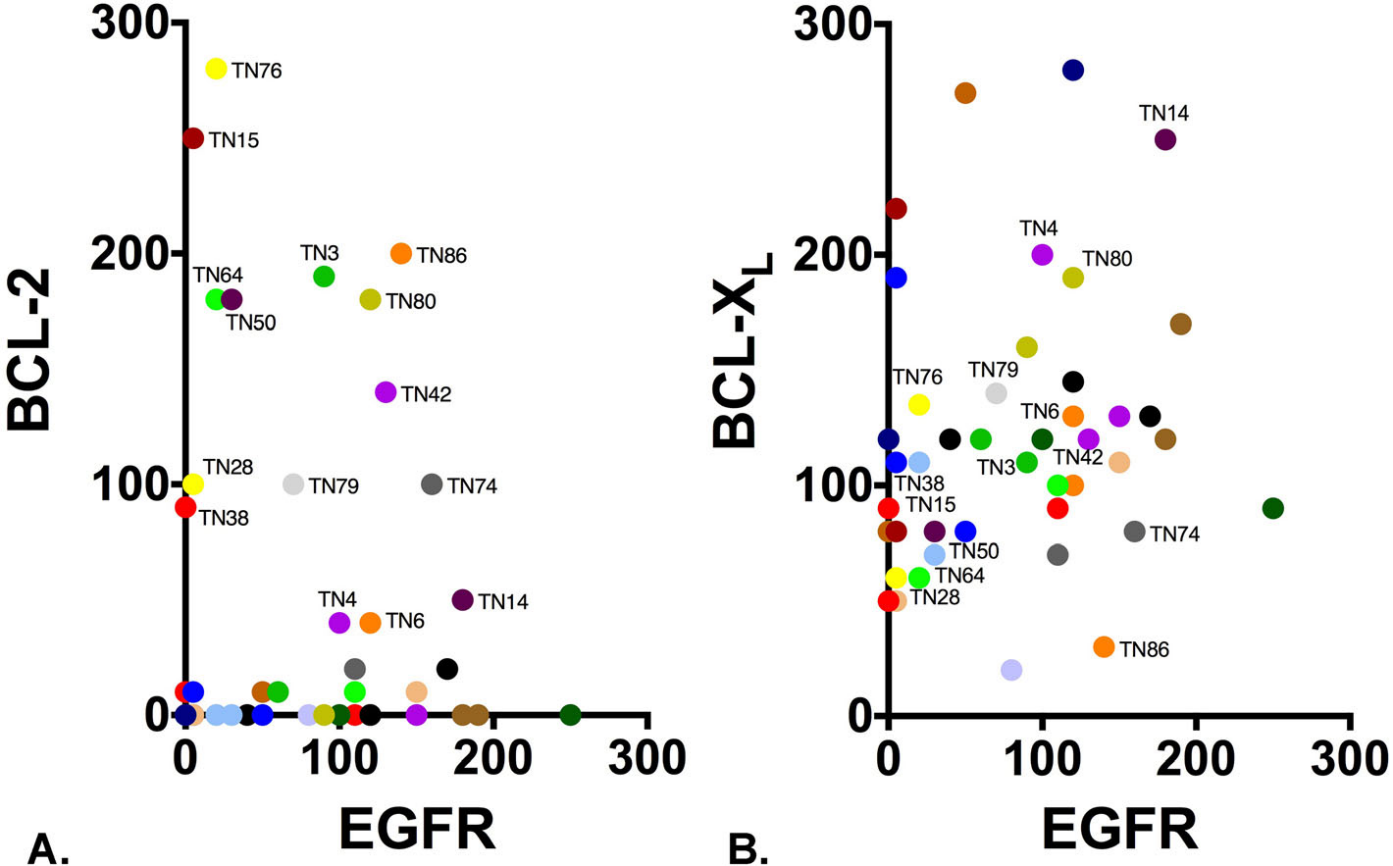
Clinical evaluation of EGFR and BCL-2/X_L_ co-expression. Forty-six triple-negative breast cancers were evaluable for EGFR, BCL-2, and BCL-X_L_ co-expression analysis. The graphs depict the co-expression of either BCL-2:EGFR (**a**) or BCL-X_L_:EGFR (**b**). Each symbol represents an individual clinical case (TN#). Each case is colored and or labeled for comparison purposes. *H*-scores [0–300] are presented for EGFR and BCL-2/X_L_

Our pre-clinical results provide evidence that navitoclax enhances the effectiveness of EGFR-targeted ADCs and highlight the potential clinical utility of navitoclax+ABBV-321 as an effective treatment option for EGFR-expressing TNBC. To evaluate the co-expression of EGFR and BCL-2/X_L_ within patient triple-negative tumors, we utilized a human tumor microarray (HTMA-240) comprising triple-negative breast cancers. Forty-six TNBC cases (TN#) arrayed on HTMA-240 were evaluable for EGFR, BCL-2, and BCL-X_L_. To compare EGFR expression levels within these tumors, we performed IHC assays and *H*-score (*H*) assessment as described above (Fig. 1). EGFR expression (*H* > 0) was detected in 87% of tumors (*n* = 40), and the majority of these cases were characterized by EGFR *H*-scores ≥ 50 (*n* = 27) (Fig. 6). Co-expression of the pro-survival proteins BCL-2 (Fig. 6a) and BCL-X_L_ (Fig. 6b) was also characterized for these tumors. Only 28% of tumors evaluated were distinguished by BCL-2 expression greater than an *H*-score of 50 (*n* = 13); the majority either expressed low levels (0 < *H* < 50; *n* = 10) or undetectable levels (*H* = 0; *n* = 23) of BCL-2. Compared to BCL-2, BCL-X_L_ expression was detected in 100% of tumors (*n* = 46), and the majority of these cases were characterized by BCL-X_L_ *H*-scores ≥ 50 (*n* = 44). Although comparative assessment of BCL-2 and BCL-X_L_ (Fig. 6) revealed predominant BCL-X_L_ expression within tumors (BCL-X_L_ > BCL-2; *n* = 35), the subset of tumors distinguished by notable BCL-2 expression co-expressed BCL-X_L_ at levels less than BCL-2 (BCL-X_L_ < BCL-2; *n* = 7) or comparable to BCL-2 (BCL-X_L_ ≈ BCL-2; *n* = 4).

## Discussion

Here, we describe the treatment responses of multiple EGFR-expressing triple-negative patient-derived xenografts to drug combinations that included an EGFR-targeted ADC together with navitoclax. Navitoclax enhanced the effectiveness of the cytotoxic payloads delivered via either ABT-414 or ABBV-321 and resulted in tumor regressions and pathological responses. Beyond efficacy assessment, we also determined that the majority of triple-negative patient tumors expressed EGFR and co-expressed BCL-X_L_ or both BCL-2 and BCL-X_L_. These data support further evaluation of combined targeting of EGFR and BCL-2/X_L_ for the treatment of triple-negative patient tumors. Together with our previous studies demonstrating dramatic combination efficacy of navitoclax+T-DM1 [15], these results provide strong support for a translational paradigm involving antibody-drug conjugates together with anti-apoptotic inhibitors in order to ameliorate the systemic toxicities associated with these therapies [33].

The PDX models employed here recapitulate several relevant clinical scenarios of TNBC [16]. Multiple models were derived from primary breast tumors without prior treatment exposure and other models were derived from metastatic breast cancers with multiple prior treatment exposures. Importantly, the two PDX models evaluated for and defined by navitoclax+ABBV-321 treatment responses (HCI-010; HCI-025) were derived from treatment-exposed patients with advanced/metastatic TNBC. HCI-010 was established from a pleural effusion following clinical exposures to five prior treatments, whereas HCI-025 was established from a cutaneous metastasis following clinical exposures to nine prior treatments. Similar to triple-negative patient tumors [9], the triple-negative PDXs evaluated here were characterized by low-level *EGFR* copy number alterations and a range of EGFR expression levels. The significant tumor regressions and pathological responses achieved, following combined treatments of the tumors that displayed the highest expression of EGFR and the highest percentage of tumor cells with ≥ 4 *EGFR* copies (HCI-010 and HCI-025), suggest that navitoclax+ABBV-321 would be an effective treatment option for previously treated advanced/metastatic TNBC that express high levels of EGFR. Furthermore, the navitoclax sensitivities observed in the HCI-010 model, which was characterized by low-level BCL-2/X_L_ expression, suggest that low levels are functionally relevant and that efficacy of combined treatments does not require high levels of BCL-2/X_L_. Our results also demonstrated that ABBV-321 is more effective than ABT-414 in these models. This is likely due to the higher affinity of the EGFR antibody associated with ABBV-321 compared to ABT-414 as well as the differential potency and or mechanism of action of PBD relative to MMAF.

One notable observation is the more pronounced navitoclax+ABBV-321 responses in HCI-010 compared to HCI-025 tumors. Despite comparable *EGFR* copy number alterations and EGFR expression levels, HCI-010 tumors treated with navitoclax+ABBV-321 achieved near-complete tumor regressions and pathological responses. Interestingly, HCI-010 carries a *BRCA1* mutation. This mutation prevents homologous repair (HR) of DNA double-strand breaks (DSB) [34, 35], and confers sensitivities to DNA alkylating agents [36] and PARP-inhibitors [37]. Consistent with our observations and perhaps explaining enhanced ABBV-321 responses in HCI-010 tumors, recent studies have associated *BRCA1/2* mutations with sensitivities to DNA-damaging PBD-containing antibody-drug conjugates [38]. Given that 10% of triple-negative breast cancers harbor *BRCA1/2* mutations [39], our results support consideration of the PBD-containing EGFR-targeted ADC (ABBV-321) within this specific subset of EGFR-expressing TNBC.

Treatment with AB095-PBD (non-tumor targeted antibody-drug conjugate) alone, and in combination with navitoclax, caused a reduction in tumor volume within both PDX models tested; however, ABBV-321 responses were significantly greater, supporting EGFR-mediated effects. Moreover, in contrast to ABBV-321, AB095-PBD treatment of HCI-010 and HCI-025 tumor cells in vitro failed to induce mitochondrial apoptotic signaling, as measured by DBP, suggesting that AB095-PBD responses were restricted to tumors in vivo. As indicated above, these observations are consistent with previous reports of antigen-independent ADC accumulation and uptake within pre-clinical tumor models [25, 29, 32], and likely due to enhanced permeability and retention (EPR) effects in vivo [24]. While vascular leakiness underscores intra-tumor drug accumulation via EPR [40], antigen-independent uptake mechanisms have been linked to microenvironment-associated ADC linker stabilities [29] and with enhanced tumor macropinocytosis at the cellular level [25–28]. The differential AB095-PBD sensitivities observed within HCI-010 and HCI-025 suggests that tumors have variable propensities for antigen-independent ADC accumulation and uptake.

Mortalities were observed in a subset of HCI-010 and HCI-025 tumor-bearing mice treated with combined agents (navitoclax + PBD-loaded ADCs). These events were largely unrelated to clear signs of toxicity, as measured by body weight reductions, or efficacy as measured by tumor volume. Since ABBV-321 does not cross-react with murine EGFR and mortalities were also observed with combined AB095-PBD treatments, these events are considered unrelated to any EGFR-mediated side effects on normal tissues. Although safety and tolerability of these agents will require advanced pharmacological and toxicological assessments in models more predictive of human physiology, several toxicities (e.g., myelosuppression, nephrotoxicity, hepatotoxicity) have been reported with PBD-containing ADCs in animals and patients [41], whereas thrombocytopenia is a known side effect associated with navitoclax [42, 43]. Importantly, these types of adverse events are clinically manageable and avoidable. For example, liver function can be readily assessed via measurements of alanine transaminase (ALT) and aspartate aminotransferase (AST), and treatment schedules can be adapted accordingly whereas navitoclax-associated thrombocytopenia can be assessed during routine blood labs and mitigated via pulsatile treatments [15]. Interestingly, recent pre-clinical studies have demonstrated that fractionated dosing of PBD-containing ADCs mitigates side effects and maintains effectiveness [44]. Although the ABBV-321 recommended clinical dosing regimen remains to be established, previous clinical testing of other PBD-loaded ADCs indicates that clinical doses have been much lower than used here, and or at much longer intervals for a very limited number of cycles [45–47]. Clinical dosing, safety, and tolerability will be ultimately informed by results from an ongoing phase I clinical trial investigating ABBV-321 in EGFR-overexpressing solid tumors (NCT#03234712).

Clinical studies of EGFR-targeted therapies within the context of breast cancer have been discouraging [1, 10]; however, these trials were largely designed without selection for and treatment of EGFR-expressing triple-negative patient tumors. These trials were also designed with the assumption that breast cancers were EGFR dependent and without consideration of EGFR-associated adaptive response mechanisms. Nonetheless, EGFR-targeted therapies were often additionally confounded by dose-limiting EGFR-associated toxicities, which most likely compromised efficient disruption of EGFR signaling. Thus, it remained unclear whether EGFR is an actionable and relevant target and whether it can be exploited safely. The efficacy of ABBV-321 suggests EGFR is actionable and relevant within the context of TNBC. This agent enables tumor-specific EGFR-targeting as well as subsequent delivery of the cytotoxic payload independent of EGFR dependency. While its efficacy in combination with navitoclax was highly efficacious, single-agent activity could also be explored clinically.

In summary, the tumor regressions and pathological responses achieved with navitoclax + EGFR-targeted ADCs, together with the finding that the majority of TNBCs co-express EGFR and BCL-2/X_L_ underscore the significant potential of navitoclax to enhance the effectiveness of EGFR-targeting ADCs and highlight navitoclax+ABBV-321 as a treatment option for therapy-resistant advanced/metastatic TNBCs that express high levels of EGFR. Additional studies are required to understand the safety of this combination.

## Conclusions

Breast cancers classified as triple-negative account for 15–20% of breast tumors and are characterized by aggressive clinical courses and overall poor prognoses. Treatment options for advanced/metastatic TNBC, which have progressed on prior treatments, are ineffective and limited. As a result, many patients experience disease progression and succumb to their disease. Thus, treatment advancements are essential for improving the care and management of TNBC and for eliminating the mortalities associated with metastatic disease. Here, we identified a novel and effective combined treatment strategy for EGFR-expressing TNBC. Employing clinically relevant patient-derived xenografts of EGFR-expressing advanced/metastatic TNBC, we provide pre-clinical evidence that navitoclax enhances the effectiveness of EGFR-targeted ADCs. Our pre-clinical results provide a strong rationale for the translational consideration of combined navitoclax and EGFR-targeted ADC treatments within the context of EGFR-expressing TNBC. Moving this combination of drugs into the clinic will require careful evaluation of treatment doses and schedules in order to minimize toxicities.

## Supplementary Information

**Additional file 1:** Supplementary tables and figures.

## Data Availability

Not applicable.

## Abbreviations

ADC: Antibody-drug conjugate
ALT: Alanine transaminase
AST: Aspartate aminotransferase
DBP: Dynamic BH3 profiling
BWH: Brigham and Women’s Hospital
DFCI: Dana-Farber Cancer Institute
DSB: DNA double-strand breaks
EGFR: Epidermal growth factor receptor
EGFRvIII: EGFR variant III
EPR: Enhanced permeability and retention
ER: Estrogen receptor
FFPE: Formalin-fixed paraffin-embedded
*h*: Height
*H*: *H*-score
*H*_*x̅*_: Average *H*-score
HCC: Harvard Cancer Center
HCI: Huntsman Cancer Institute
H&E: Hematoxylin and eosin
HER2: Human epidermal growth factor receptor-2
HR: Homologous repair
IHC: Immunohistochemistry
FISH: Fluorescence in situ hybridization
i.p.: Intraperitoneal
*l*: Length
mc: Maleimidocaproyl
mc-VC: Maleimidocaproyl-valine-citrulline
MMAE: Monomethyl auristatin E
MMAF: Monomethyl auristatin F
MT: Metastatic breast tumor
*n*: Number
NS: Non-significant
NT-NOD.scid: Nontumor-bearing NOD.scid
PARP: Poly (ADP-ribose) polymerase
PBD: Pyrrolobenzodiazepine
PDX: Patient-derived xenograft
p.o.: Per os
PR: Progesterone receptor
PE: Pleural effusion
PT: Primary breast tumor
TMA: Tissue microarray
TNBC: Triple-negative breast cancer
T×: Treatment
*w*: Width

## References

[1] Masuda H, Zhang D, Bartholomeusz C, Doihara H, Hortobagyi GN, Ueno NT (2012). Role of epidermal growth factor receptor in breast cancer. Breast Cancer Res Treat.

[2] Rakha EA, El-Sayed ME, Green AR, Lee AH, Robertson JF, Ellis IO (2007). Prognostic markers in triple-negative breast cancer. Cancer..

[3] Tan DS, Marchio C, Jones RL, Savage K, Smith IE, Dowsett M (2008). Triple negative breast cancer: molecular profiling and prognostic impact in adjuvant anthracycline-treated patients. Breast Cancer Res Treat.

[4] Viale G, Rotmensz N, Maisonneuve P, Bottiglieri L, Montagna E, Luini A (2009). Invasive ductal carcinoma of the breast with the “triple-negative” phenotype: prognostic implications of EGFR immunoreactivity. Breast Cancer Res Treat.

[5] Martin V, Botta F, Zanellato E, Molinari F, Crippa S, Mazzucchelli L (2012). Molecular characterization of EGFR and EGFR-downstream pathways in triple negative breast carcinomas with basal like features. Histol Histopathol.

[6] Meseure D, Vacher S, Drak Alsibai K, Trassard M, Susini A, Le Ray C (2012). Profiling of EGFR mRNA and protein expression in 471 breast cancers compared with 10 normal tissues: a candidate biomarker to predict EGFR inhibitor effectiveness. Int J Cancer.

[7] Liu D, He J, Yuan Z, Wang S, Peng R, Shi Y (2012). EGFR expression correlates with decreased disease-free survival in triple-negative breast cancer: a retrospective analysis based on a tissue microarray. Med Oncol.

[8] Choi J, Jung WH, Koo JS (2012). Clinicopathologic features of molecular subtypes of triple negative breast cancer based on immunohistochemical markers. Histol Histopathol.

[9] Park HS, Jang MH, Kim EJ, Kim HJ, Lee HJ, Kim YJ (2014). High EGFR gene copy number predicts poor outcome in triple-negative breast cancer. Mod Pathol.

[10] Nakai K, Hung MC, Yamaguchi H (2016). A perspective on anti-EGFR therapies targeting triple-negative breast cancer. Am J Cancer Res.

[11] Reilly EB, Phillips AC, Buchanan FG, Kingsbury G, Zhang Y, Meulbroek JA (2015). Characterization of ABT-806, a humanized tumor-specific anti-EGFR monoclonal antibody. Mol Cancer Ther.

[12] Phillips AC, Boghaert ER, Vaidya KS, Mitten MJ, Norvell S, Falls HD (2016). ABT-414, an antibody-drug conjugate targeting a tumor-selective EGFR epitope. Mol Cancer Ther.

[13] Phillips AC, Boghaert ER, Vaidya KS, Falls HD, Mitten MJ, DeVries PJ (2018). Characterization of ABBV-221, a tumor-selective EGFR-targeting antibody drug conjugate. Mol Cancer Ther.

[14] Anderson MG, Falls HD, Mitten MJ, Oleksijew A, Vaidya KS, Boghaert ER, et al. Targeting multiple EGFR expressing tumors with a highly potent tumor-selective antibody drug conjugate. Mol Cancer Ther. 2020;19(10):2117–25. 10.1158/1535-7163.MCT-20-0149 32847977

[15] Zoeller JJ, Vagodny A, Taneja K, Tan BY, O’Brien N, Slamon DJ (2019). Neutralization of BCL-2/XL enhances the cytotoxicity of T-DM1 in vivo. Mol Cancer Ther.

[16] DeRose YS, Wang G, Lin YC, Bernard PS, Buys SS, Ebbert MT (2011). Tumor grafts derived from women with breast cancer authentically reflect tumor pathology, growth, metastasis and disease outcomes. Nat Med.

[17] DeRose YS, Gligorich KM, Wang G, Georgelas A, Bowman P, Courdy SJ, et al. Patient-derived models of human breast cancer: protocols for in vitro and in vivo applications in tumor biology and translational medicine. Curr Protoc Pharmacol. 2013;Chapter 14:Unit14 23. 10.1002/0471141755.ph1423s60 PMC3630511 23456611

[18] Cappuzzo F, Hirsch FR, Rossi E, Bartolini S, Ceresoli GL, Bemis L (2005). Epidermal growth factor receptor gene and protein and gefitinib sensitivity in non-small-cell lung cancer. J Natl Cancer Inst.

[19] Ogston KN, Miller ID, Payne S, Hutcheon AW, Sarkar TK, Smith I (2003). A new histological grading system to assess response of breast cancers to primary chemotherapy: prognostic significance and survival. Breast..

[20] Zoeller JJ, Bronson RT, Selfors LM, Mills GB, Brugge JS. Niche-localized tumor cells are protected from HER2-targeted therapy via upregulation of an anti-apoptotic program in vivo. npj Breast Cancer. 2017;3(18):1–7. 10.1038/s41523-017-0020-z PMC5460247 28649658

[21] Montero J, Sarosiek KA, DeAngelo JD, Maertens O, Ryan J, Ercan D (2015). Drug-induced death signaling strategy rapidly predicts cancer response to chemotherapy. Cell..

[22] Bhola PD, Ahmed E, Guerriero JL, Sicinska E, Su E, Lavrova E, et al. High-throughput dynamic BH3 profiling may quickly and accurately predict effective therapies in solid tumors. Sci Signal. 2020;13(636):1–11. 10.1126/scisignal.aay1451 PMC8023011 32546544

[23] Blot V, Richardson R, Coronella J. The New Generation of Antibody Drug Conjugates. Forum on Immunopathological Dis Ther. 2014;5(1–2):25–45.

[24] Matsumura Y, Maeda H (1986). A new concept for macromolecular therapeutics in cancer chemotherapy: mechanism of tumoritropic accumulation of proteins and the antitumor agent smancs. Cancer Res.

[25] Jedema I, Barge RM, van der Velden VH, Nijmeijer BA, van Dongen JJ, Willemze R (2004). Internalization and cell cycle-dependent killing of leukemic cells by gemtuzumab ozogamicin: rationale for efficacy in CD33-negative malignancies with endocytic capacity. Leukemia..

[26] Ha KD, Bidlingmaier SM, Liu B (2016). Macropinocytosis exploitation by cancers and cancer therapeutics. Front Physiol.

[27] Zhao H, Gulesserian S, Ganesan SK, Ou J, Morrison K, Zeng Z (2017). Inhibition of megakaryocyte differentiation by antibody-drug conjugates (ADCs) is mediated by macropinocytosis: implications for ADC-induced thrombocytopenia. Mol Cancer Ther.

[28] Zhao H, Atkinson J, Gulesserian S, Zeng Z, Nater J, Ou J (2018). Modulation of macropinocytosis-mediated internalization decreases ocular toxicity of antibody-drug conjugates. Cancer Res.

[29] Boghaert ER, Khandke K, Sridharan L, Armellino D, Dougher M, Dijoseph JF (2006). Tumoricidal effect of calicheamicin immuno-conjugates using a passive targeting strategy. Int J Oncol.

[30] Takakura Y, Fujita T, Hashida M, Sezaki H (1990). Disposition characteristics of macromolecules in tumor-bearing mice. Pharm Res.

[31] Karanikas G, Ulrich-Pur H, Becherer A, Wiesner K, Dudczak R, Raderer M (2002). Uptake of indium-111-labeled human polyclonal immunoglobulin G in pancreatic cancer: in vivo and in vitro studies. Oncol Rep.

[32] Boghaert ER, Sridharan L, Armellino DC, Khandke KM, DiJoseph JF, Kunz A (2004). Antibody-targeted chemotherapy with the calicheamicin conjugate hu3S193-N-acetyl gamma calicheamicin dimethyl hydrazide targets Lewisy and eliminates Lewisy-positive human carcinoma cells and xenografts. Clin Cancer Res.

[33] Vlahovic G, Karantza V, Wang D, Cosgrove D, Rudersdorf N, Yang J (2014). A phase I safety and pharmacokinetic study of ABT-263 in combination with carboplatin/paclitaxel in the treatment of patients with solid tumors. Investig New Drugs.

[34] Moynahan ME, Chiu JW, Koller BH, Jasin M (1999). Brca1 controls homology-directed DNA repair. Mol Cell.

[35] Tutt A, Bertwistle D, Valentine J, Gabriel A, Swift S, Ross G (2001). Mutation in Brca2 stimulates error-prone homology-directed repair of DNA double-strand breaks occurring between repeated sequences. EMBO J.

[36] Bhattacharyya A, Ear US, Koller BH, Weichselbaum RR, Bishop DK (2000). The breast cancer susceptibility gene BRCA1 is required for subnuclear assembly of Rad51 and survival following treatment with the DNA cross-linking agent cisplatin. J Biol Chem.

[37] Farmer H, McCabe N, Lord CJ, Tutt AN, Johnson DA, Richardson TB (2005). Targeting the DNA repair defect in BRCA mutant cells as a therapeutic strategy. Nature..

[38] Zhong H, Chen C, Tammali R, Breen S, Zhang J, Fazenbaker C (2019). Improved therapeutic window in BRCA-mutant tumors with antibody-linked pyrrolobenzodiazepine dimers with and without PARP inhibition. Mol Cancer Ther.

[39] Hartman AR, Kaldate RR, Sailer LM, Painter L, Grier CE, Endsley RR (2012). Prevalence of BRCA mutations in an unselected population of triple-negative breast cancer. Cancer..

[40] Tabrizi M, Bornstein GG, Suria H (2010). Biodistribution mechanisms of therapeutic monoclonal antibodies in health and disease. AAPS J.

[41] Saber H, Simpson N, Ricks TK, Leighton JK (2019). An FDA oncology analysis of toxicities associated with PBD-containing antibody-drug conjugates. Regul Toxicol Pharmacol.

[42] Zhang H, Nimmer PM, Tahir SK, Chen J, Fryer RM, Hahn KR (2007). Bcl-2 family proteins are essential for platelet survival. Cell Death Differ.

[43] Tse C, Shoemaker AR, Adickes J, Anderson MG, Chen J, Jin S (2008). ABT-263: a potent and orally bioavailable Bcl-2 family inhibitor. Cancer Res.

[44] Hinrichs MJM, Ryan PM, Zheng B, Afif-Rider S, Yu XQ, Gunsior M (2017). Fractionated dosing improves preclinical therapeutic index of pyrrolobenzodiazepine-containing antibody drug conjugates. Clin Cancer Res.

[45] Morgensztern D, Besse B, Greillier L, Santana-Davila R, Ready N, Hann CL (2019). Efficacy and safety of rovalpituzumab tesirine in third-line and beyond patients with DLL3-expressing, relapsed/refractory small-cell lung cancer: results from the phase II TRINITY Study. Clin Cancer Res.

[46] Phillips T, Barr PM, Park SI, Kolibaba K, Caimi PF, Chhabra S (2019). A phase 1 trial of SGN-CD70A in patients with CD70-positive diffuse large B cell lymphoma and mantle cell lymphoma. Investig New Drugs.

[47] Jain N, Stock W, Zeidan A, Atallah E, McCloskey J, Heffner L (2020). Loncastuximab tesirine, an anti-CD19 antibody-drug conjugate, in relapsed/refractory B-cell acute lymphoblastic leukemia. Blood Adv.

