# Supplementary material for: Navitoclax enhances the effectiveness of EGFR-targeted antibody-drug conjugates in PDX models of EGFR-expressing triple-negative breast cancer
Source: Breast Cancer Res. 2020 Nov 30;22:132. doi: 10.1186/s13058-020-01374-8 (PMC7708921; doi:10.1186/s13058-020-01374-8)
Supplement: Supplementary file 1 — Additional file 1: Supplementary tables and figures. [file 13058_2020_1374_MOESM1_ESM.pdf]

SUPPLEMENTAL TABLE 1. *EGFR* FISH-based copy number assessments

Supplemental Table 1A.

Classification	Abbreviation	Description[1]
disomic	DIS	⩽ 2 copies in > 90% of cells
lo trisomic	LO-TRI	⩽ 2 copies in ⩾ 40% of cells AND 3 copies in 10-40% of cells AND ⩾ 4 copies in < 10% of cells
hi trisomic	HI-TRI	⩽ 2 copies in ⩾ 40% of cells AND 3 copies in ⩾ 40% of cells AND ⩾ 4 copies in < 10% of cells
lo polysomic	LO-POL	⩾ 4 copies in 10-40% of cells
hi polysomic	HI-POL	⩾ 4 copies in ⩾ 40% of cells
amplification	AMP	tight *EGFR* gene clusters AND *EGFR*:CEP7 ⩾ 2 OR ⩾ 15 copies in ⩾ 10% of cells

Supplemental Table 1B.

PDX[2]	Number of Cells	Total *EGFR* for N cells	Total CEP7 for N cells	Average *EGFR*/cell	Average CEP7/cell	Ratio *EGFR*/CEP7	⩾ 4 *EGFR* per cell (%)	FISH classification	EGFR H-score[3]	3+ EGFR %
**HCI-001**	30	67.2	91.3	2.24	3.04	0.74	3.3	LO-TRI	**188**	26
**HCI-002**	30	71.3	70.3	2.38	2.34	1.01	0.6	LO-TRI	**121**	16
**HCI-004**	20	41.0	38.2	2.05	1.91	1.07	2.0	LO-TRI	**76**	0
**HCI-010**	30	89.0	87.3	2.97	2.91	1.02	25.8	LO-POL	**228**	38
**HCI-015**	30	67.3	81.5	2.24	2.72	0.83	2.5	LO-TRI	**193**	33
**HCI-016**	30	75.8	67.3	2.53	2.24	1.13	10.0	HI-TRI	**90**	0
**HCI-019**	30	71.5	66.8	2.38	2.23	1.07	5.8	LO-TRI	**208**	19
**HCI-025**	30	76	70	2.53	2.33	1.09	16.7	LO-POL	**230**	30

[1]FISH-based copy number classifiers as described (47)
[2]*EGFR*:CEP7 FISH values represent either the average of 4-6 tumors (HCI-001;002;004;010;015;016;019) or one tumor (HCI-025) evaluated
[3]EGFR H-scores as determined in **Table 2** and **Supplemental Table 3**

SUPPLEMENTAL TABLE 2. Figure-associated p-values

Supplemental Table 2A.

PDX	HCI-001	HCI-002	HCI-004	HCI-010	HCI-015	HCI-016	HCI-019
Figure 2A.	0.0038	< 0.0001	0.0105	0.0011	0.0786	0.3159	0.0090

Supplemental Table 2B.

HCI-010		vehicles	263	414	263+414	095	263+095
Figure 3A.	**vehicles**	NA	0.0075	0.1676	0.0007	0.2707	0.0047

Supplemental Table 2C.

HCI-010		vehicles	263	321	263+321	095	263+095
Figure 4A.	**vehicles**	NA	0.0006	< 0.0001	< 0.0001	0.0011	< 0.0001
**HCI-025**		**vehicles**	**263**	**321**	**263+321**	**095**	**263+095**
Figure 4C.	**vehicles**	NA	0.0143	< 0.0001	< 0.0001	< 0.0001	< 0.0001

Supplemental Table 2D.

HCI-010		vehicles	263	321	263+321	095	263+095
Figure 5A.	**vehicles**	NA	0.0004	0.0080	< 0.0001	0.1723	0.0007
**HCI-025**		**vehicles**	**263**	**321**	**263+321**	**095**	**263+095**
Figure 5B.	**vehicles**	NA	0.1280	0.0223	0.0056	0.3973	0.4740

Supplemental Table 2E.

PDX	HCI-001	HCI-002	HCI-004	HCI-010	HCI-015	HCI-016	HCI-019
Supplemental Figure 2B.	0.0439	< 0.0001	0.0005	0.0148	0.4633	0.1789	0.2968

Supplemental Table 2F.

HCI-010		vehicles	263	414	263+414	095	263+095
Supplemental Figure 3B.	**vehicles**	NA	0.1825	0.2469	0.0442	0.2551	0.1878

Supplemental Table 2G.

HCI-010		vehicles	263	321	263+321	095	263+095
Supplemental Figure 4B.	**vehicles**	NA	0.0119	< 0.0001	< 0.0001	0.0444	0.0003
**HCI-025**		**vehicles**	**263**	**321**	**263+321**	**095**	**263+095**
Supplemental Figure 6B.	**vehicles**	NA	0.0507	< 0.0001	< 0.0001	0.0073	< 0.0001

Supplemental Table 2H.

HCI-025		vehicles	263	321	263+321	095	263+095
Supplemental Figure 7B.	**vehicles**	NA	0.2111	0.0075	0.0037	0.1599	0.0267
Supplemental Figure 7C.	**vehicles**	NA	0.0199	0.0017	0.0009	0.0239	0.0023

Supplemental Table 2I.

HCI-010		vehicles	263	321	263+321	095	263+095
Supplemental Figure 8B.	**vehicles**	NA	0.2598	0.0015	0.0004	0.3222	0.0211
**HCI-025**		**vehicles**	**263**	**321**	**263+321**	**095**	**263+095**
Supplemental Figure 8F.	**vehicles**	NA	0.3414	0.0133	0.0002	0.4212	0.0659

SUPPLEMENTAL TABLE 3. PDX models of triple-negative breast cancers

PDX	Patient Source[4]	Patient Treatment[5]	HER2 Status[6]	ER Status	PR Status	EGFR H-score[7]	BCL-2 H-score	BCL-X_L H-score
**HCI-023**	PT	- prior T×	–	–	–	170	75	55
**HCI-024$_a$**	MT	+ prior T×	–	–	–	170	85	105
**HCI-024$_b$**	MT	+ prior T×	–	–	–	155	15	150
**HCI-025**	MT	+ prior T×	–	–	–	230	10	210
**HCI-027**	PT	+ prior T×	–	–	–	150	20	125
**HCI-028**	PE	+ prior T×	–	–	–	15	20	205
**HCI-030**	PT	- prior T×	–	–	–	200	30	105

[4]PT, primary breast tumor; PE, pleural effusion; MT, metastatic breast tumor
[5]T×, unpublished data kindly provided by A.W.
[6]HER2, ER, PR unpublished data kindly provided by A.W.
[7]H-score represents one tumor surveyed per PDX kindly provided by A.W.

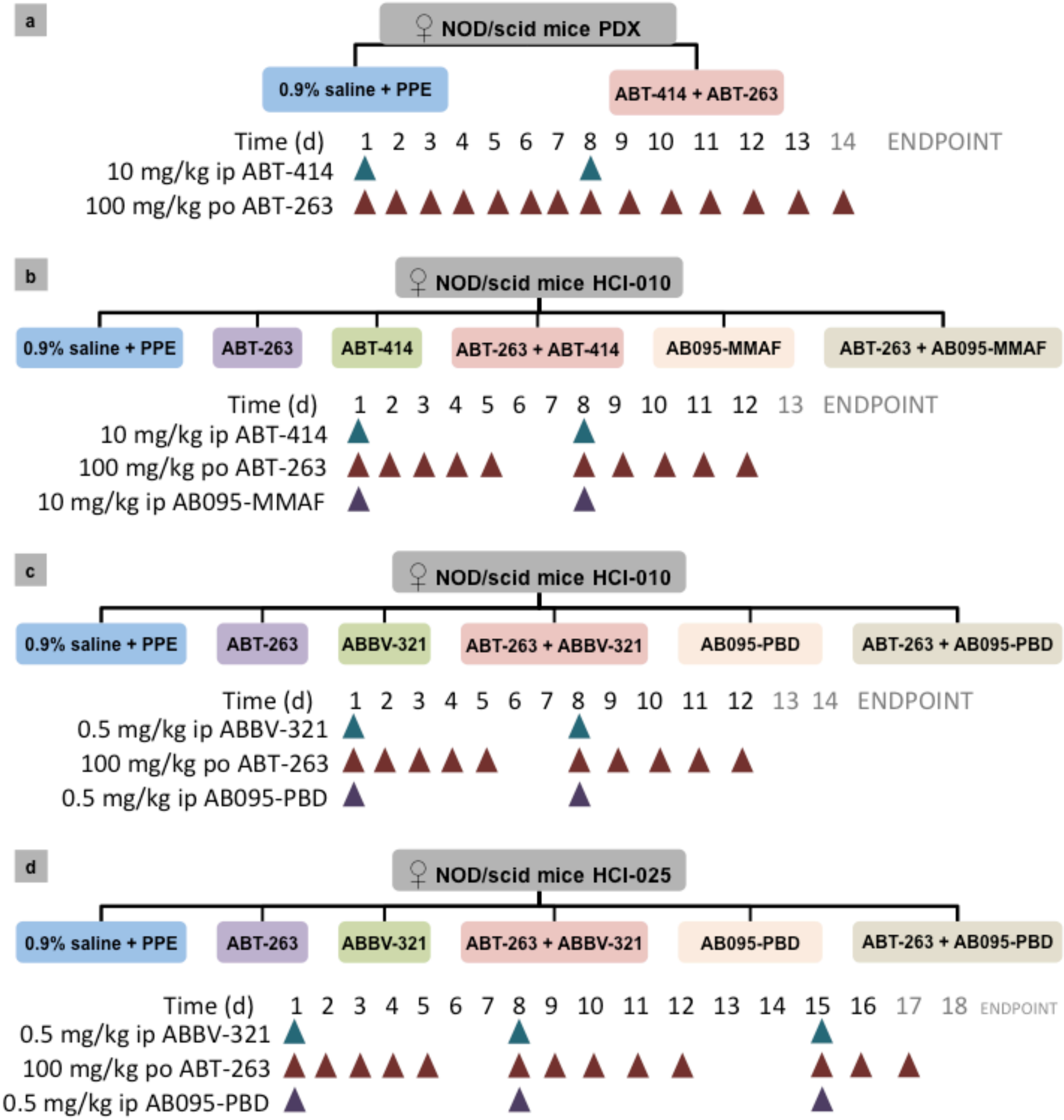

Supplemental Figure 1. Experimental designs. Treatment groups, doses and schedules are presented for **Figure 2** (**a**), **Figure 3** (**b**) and **Figure 4** (**c-d**) pre-clinical studies.

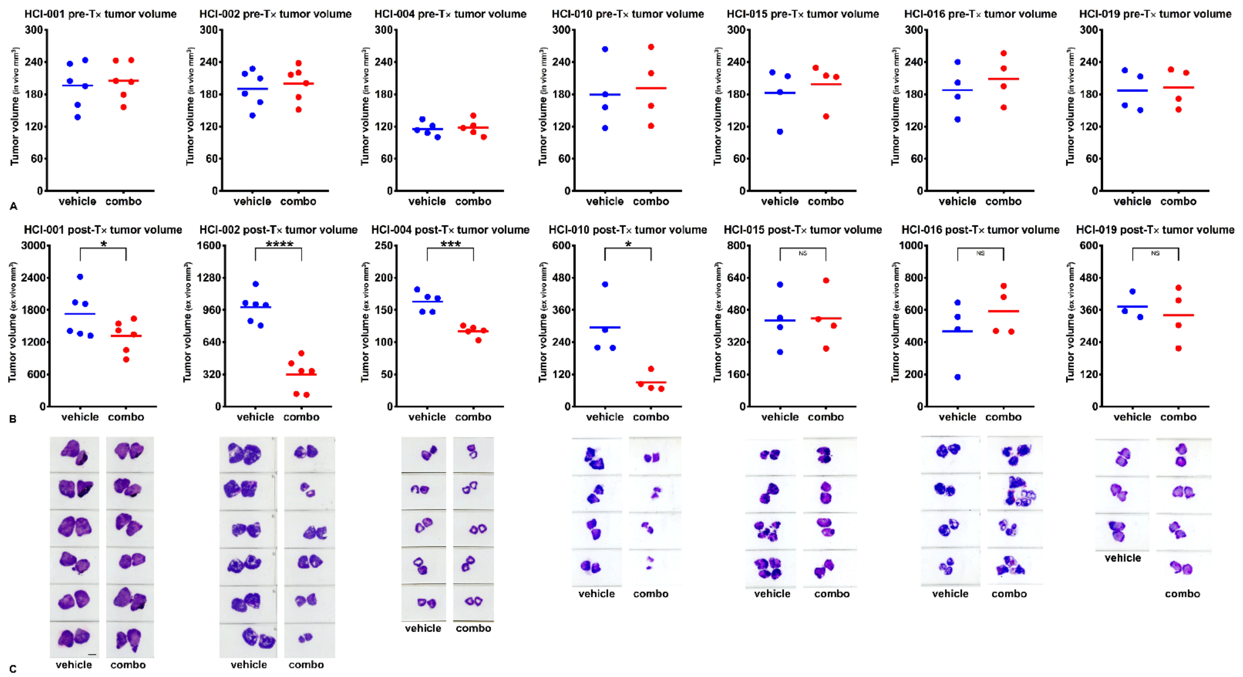

Supplemental Figure 2. PDX pre-treatment tumor volume, post-treatment tumor volume and H&E slide scans corresponding to **Figure 2**. PDX pre-treatment tumor volumes were based upon *in vivo* measurements in two dimensions (**a**). PDX post-treatment tumor volumes were based upon *ex vivo* measurements in three dimensions (**b**). Each symbol represents a tumor. Each line represents the mean. p-values < 0.05 (*), < 0.01 (**), < 0.001 (***) and < 0.0001 (****) are indicated (Welch's one-tail test). p-values > 0.05 = NS. p-values for all seven vehicle versus combo comparisons are presented in **Supplemental Table 2e**. The digitized H&E slides are presented for vehicle versus combo comparisons (**c**). Note each slide represents a tumor bisected along the longitudinal axis. Note tumor slides with more than two sections represent tumors that required trimming during the processing steps. Scale bar, ~0.5 cm.

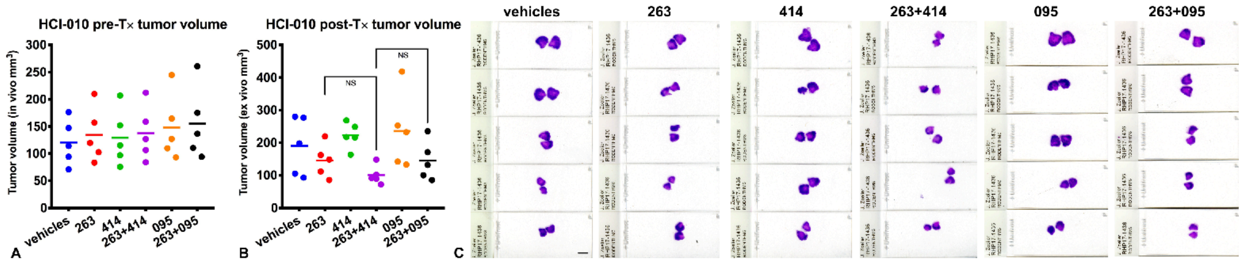

Supplemental Figure 3. HCI-010 pre-treatment tumor volume, post-treatment tumor volume and H&E slide scans corresponding to **Figure 3**. PDX pre-treatment tumor volumes were based upon *in vivo* measurements in two dimensions (**a**). PDX post-treatment tumor volumes were based upon *ex vivo* measurements in three dimensions (**b**). Each symbol represents a tumor. Each line represents the mean. p-values for the most relevant comparisons are indicated (Welch's one-tail test: ABT-263 versus ABT-263+ABT-414, p-value > 0.05 = NS and ABT-263+ABT-414 versus ABT-263+AB095-MMAF, p-value > 0.05 = NS). p-values for vehicle versus ABT-263, ABT-414, ABT-263+ABT-414, AB095-MMAF and ABT-263+AB095-MMAF comparisons are presented in **Supplemental Table 2f**. The digitized H&E slides are presented for treatment group comparisons (**c**). Note each slide represents a tumor bisected along the longitudinal axis. Scale bar, ~0.5 cm.

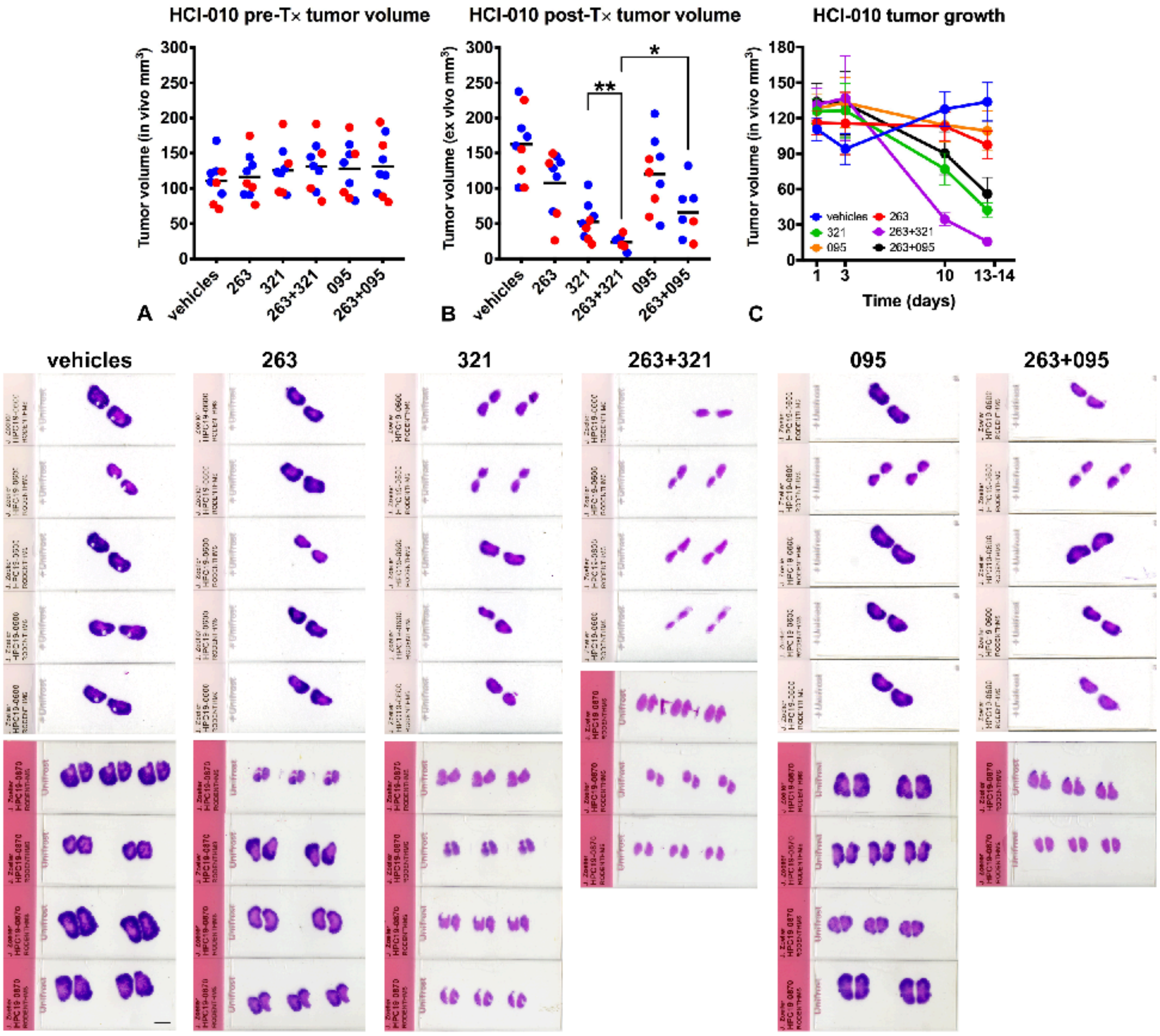

Supplemental Figure 4. HCI-010 pre-treatment tumor volume, post-treatment tumor volume, tumor growth over time and H&E slide scans corresponding to **Figure 4**. PDX pre-treatment tumor volumes were based upon *in vivo* measurements in two dimensions (**a**). PDX post-treatment tumor volumes were based upon *ex vivo* measurements in three dimensions (**b**). Each symbol represents a tumor enrolled in one of two independent treatment studies (e.g. blue, study A and red, study B). Each line represents the grand mean. p-values for the most relevant comparisons are indicated (Welch's one-tail test: p-value < 0.01 (**), ABBV-321 versus ABT-263+ABBV-321 and p-value < 0.05 (*), ABT-263+ABBV-321 versus ABT-263+AB095-PBD). p-values for vehicle versus ABT-263, ABBV-321, ABT-263+ABBV-321, AB095-PBD and ABT-263+AB095-PBD comparisons are presented in **Supplemental Table 2g**. Caliper-based assessments of tumor volume (mm^3) over time (days) were based upon *in vivo* measurements in two dimensions (**c**). Each symbol (except for d3) represents the mean of 7-9 tumors from the two independent treatment studies. d3 represents the mean of 2-4 tumors from one of the two studies. Bars, ± SEM. The digitized H&E slides are presented for treatment group comparisons (**d**). Note each slide represents a tumor bisected along the longitudinal axis. Scale bar, ~0.5 cm.

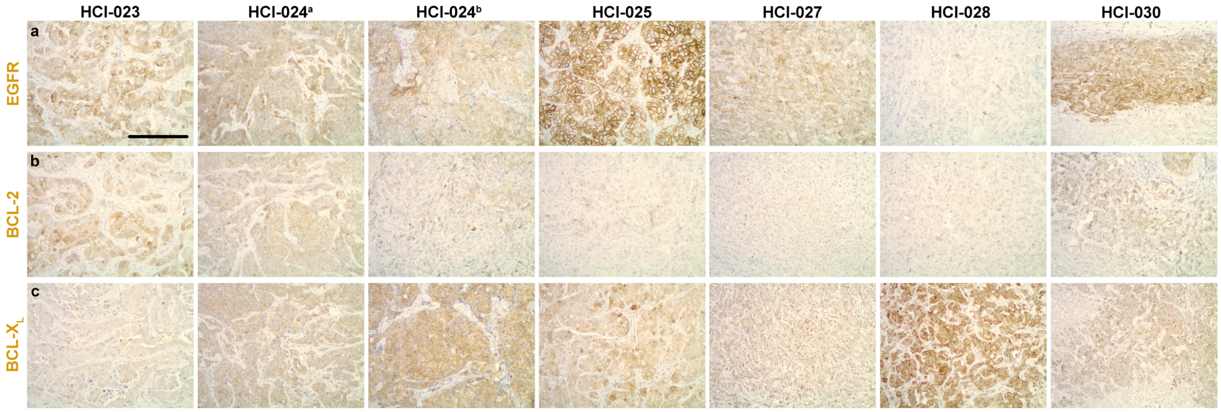

Supplemental Figure 5. Comparison of EGFR, BCL-2 and BCL-X_L expression levels. Seven additional PDX models of TNBC were immuno-stained for EGFR (**a**), BCL-2 (**b**) and BCL-X_L (**c**). Representative immuno-stains are shown. The IHC results were semi-quantitated via H-score assessment and summarized in **Supplemental Table 3**. HCI-024_a and HCI-024_b represent second-generation tumors established within two separate mice. Scale bar, ~200 µm.

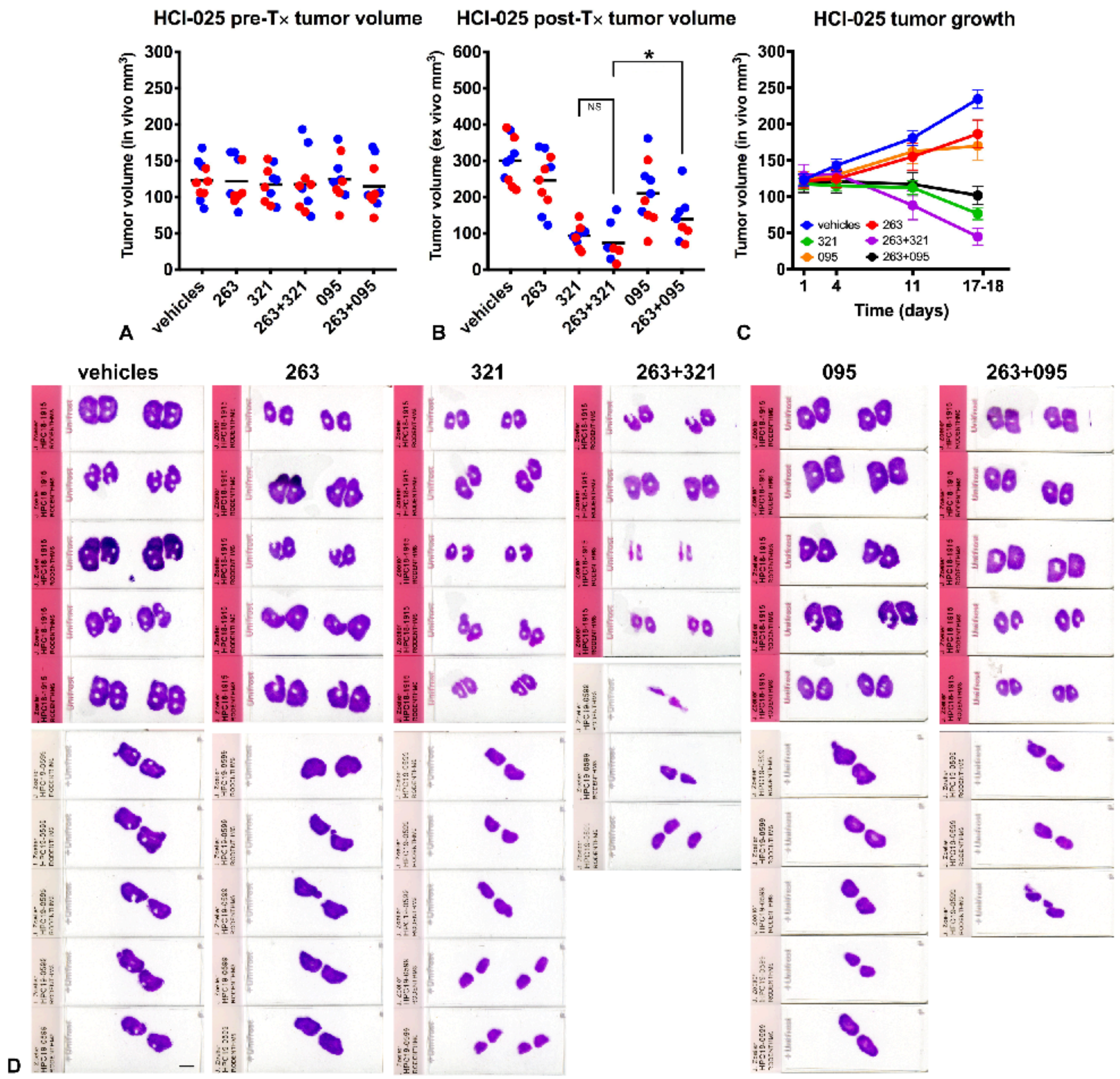

Supplemental Figure 6. HCI-025 pre-treatment tumor volume, post-treatment tumor volume, tumor growth over time and H&E slide scans corresponding to **Figure 4**. PDX pre-treatment tumor volumes were based upon *in vivo* measurements in two dimensions (**a**). PDX post-treatment tumor volumes were based upon *ex vivo* measurements in three dimensions (**b**). Each symbol represents a tumor enrolled in one of two independent treatment studies (e.g. blue, study A and red, study B). Each line represents the grand mean. p-values for the most relevant comparisons are indicated (Welch's one-tail test: p-value > 0.05 = NS, ABBV-321 versus ABT-263+ABBV-321 and p-value < 0.05 (*), ABT-263+ABBV-321 versus ABT-263+AB095-PBD). p-values for vehicle versus ABT-263, ABBV-321, ABT-263+ABBV-321, AB095-PBD and ABT-263+AB095-PBD comparisons are presented in **Supplemental Table 2g**. Caliper-based assessments of tumor volume (mm^3) over time (days) were based upon *in vivo* measurements in two dimensions (**c**). Each symbol represents the mean of 7-10 tumors from the two independent treatment studies. Bars, ± SEM. The digitized H&E slides are presented for treatment group comparisons (**d**). Note each slide represents a tumor bisected along the longitudinal axis. Scale bar, ~0.5 cm.

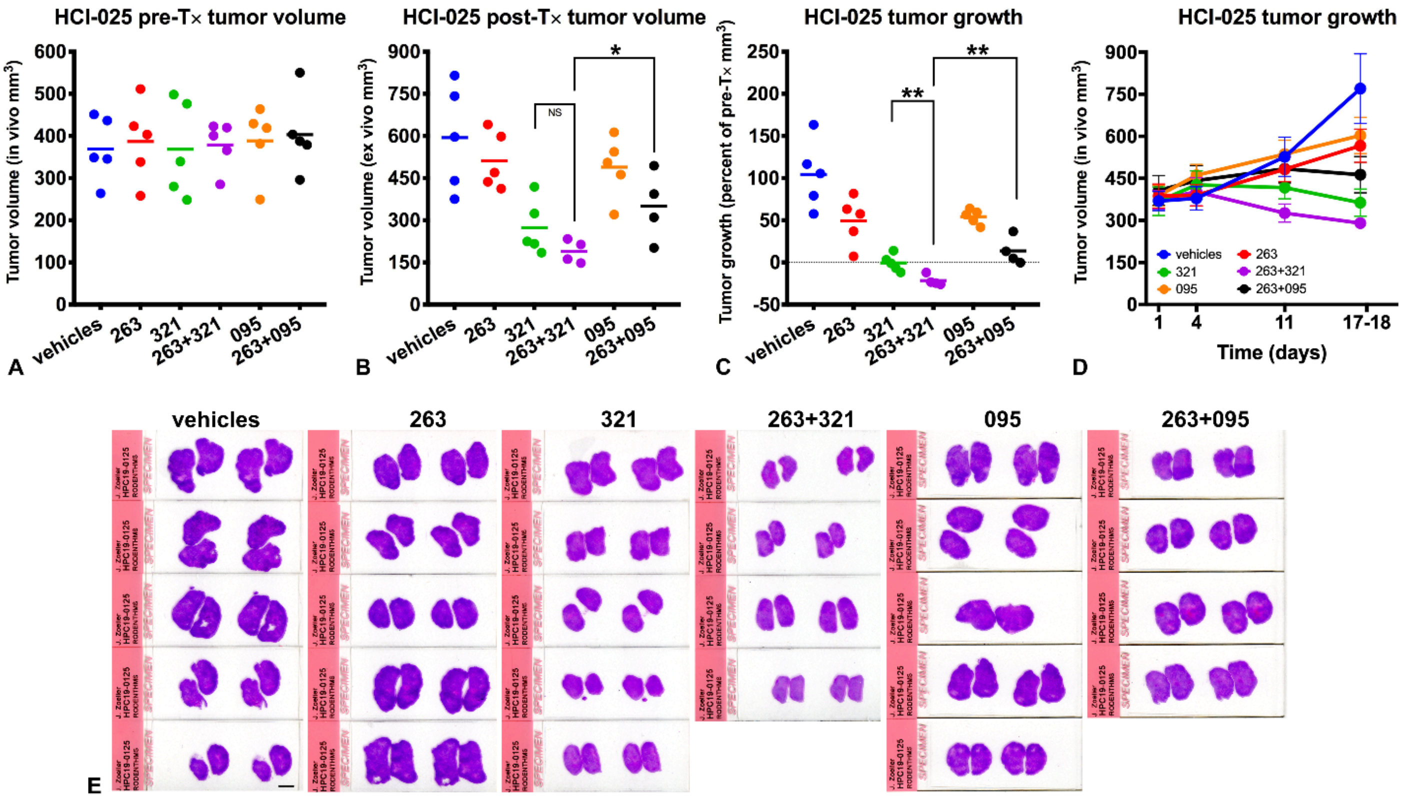

Supplemental Figure 7. HCI-025 pre-treatment tumor volume, post-treatment tumor volume, tumor growth and H&E slide scans. PDX pre-treatment tumor volumes were based upon *in vivo* measurements in two dimensions (**a**). PDX post-treatment tumor volumes were based upon *ex vivo* measurements in three dimensions (**b**). p-values for the most relevant comparisons are indicated (Welch's one-tail test: p-value > 0.05 = NS, ABBV-321 versus ABT-263+ABBV-321 and p-value < 0.05 (*), ABT-263+ABBV-321 versus ABT-263+AB095-PBD). p-values for vehicle versus ABT-263, ABBV-321, ABT-263+ABBV-321, AB095-PBD and ABT-263+AB095-PBD comparisons are presented in **Supplemental Table 2h**. Graph **c** presents tumor growth as a percent of pre-treatment tumor volume for each tumor (n = 4-5 per group). Each symbol represents a tumor. Each line represents the mean. p-values for the most relevant comparisons are indicated (Welch's one-tail test: p-value < 0.01 (**), ABBV-321 versus ABT-263+ABBV-321 and p-value < 0.01 (**), ABT-263+ABBV-321 versus ABT-263+AB095-PBD). p-values for vehicle versus ABT-263, ABBV-321, ABT-263+ABBV-321, AB095-PBD and ABT-263+AB095-PBD comparisons are presented in **Supplemental Table 2h**. Caliper-based assessments of tumor volume (mm^3) over time (days) were based upon *in vivo* measurements in two dimensions (**d**). Each symbol represents the mean of 4-5 tumors. Bars, ± SEM. The digitized H&E slides are presented for treatment group comparisons (**e**). Note each slide represents a tumor bisected along the longitudinal axis. Scale bar, ~0.5 cm.

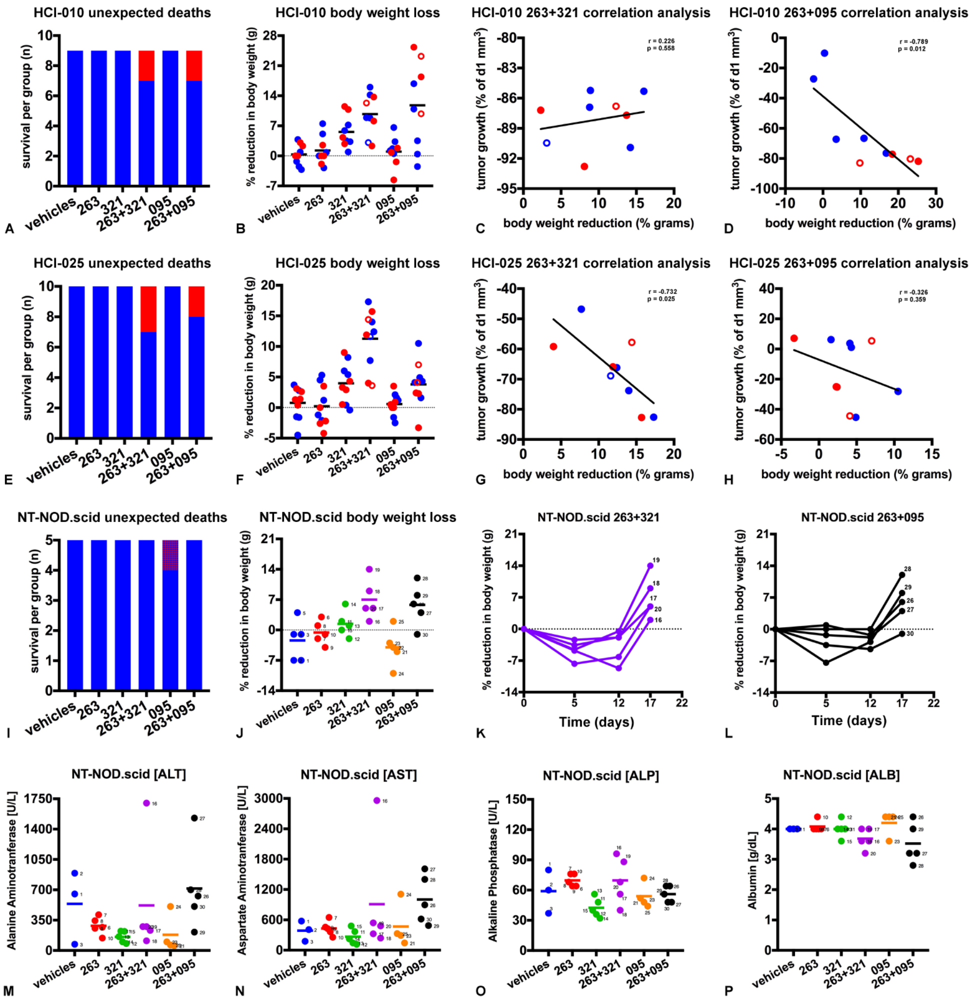

Supplemental Figure 8. Adverse events associated with combined treatments. Deaths encountered among HCI-010 (**a**) and HCI-025 (**e**) tumor-bearing mice are presented for each treatment group. Graphs **b** and **f** present body weight reductions as a percent of pre-treatment body weight for each animal. Each symbol represents an animal enrolled in one of two independent treatment studies (e.g. blue, study A and red, study, B). The open symbols highlight body weight reductions associated with deaths. Note body weight reductions associated with deaths do not represent extreme weight loss conditions. Each line represents the grand mean. p-values (Welch's one-tail test) for vehicle versus ABT-263, ABBV-321, ABT-263+ABBV-321, AB095-PBD and ABT-263+AB095-PBD comparisons are presented in **Supplemental Table 2i**. Graphs **c-d** and **g-h** compare and correlate body weight reductions and tumor volume reductions for combination-treated HCI-010 (**c-d**) and HCI-025 (**g-h**) animals. The open symbols highlight the animals that died. Note deaths were not observed in mice that exhibited extreme tumor regressions. The Pearson correlation [r] and associated p-values are indicated. Deaths encountered among nontumor-bearing mice (NT-NOD.scid) are presented for each treatment group (**i**). Note the AB095-PBD hatched-bar indicates an accidental death on day fifteen due to treatment-associated handling (#22, **j**). Graph **j** presents body weight reductions as a percent of pre-treatment body weight for each animal. Each symbol represents an animal. Each line represents the mean. Graphs **k** and **l** present body weight reductions over the course of the treatment study for PBD-loaded antibodies combined with navitoclax. Graphs **m** (alanine aminotransferase; ALT), **n** (aspartate aminotransferase; AST), **o** (alkaline phosphatase; ALP) and **p** (albumin; ALB) present serum-associated liver function testing. Each symbol represents an animal. Each line represents the mean. For cross comparisons, numerical identifiers (#1-30) are indicated for each nontumor-bearing animal. Note three serum samples were unavailable for analysis (#4, #5, #22).
